# A transcriptome-informed QSP model of metastatic triple-negative breast cancer identifies predictive biomarkers for PD-1 inhibition

**DOI:** 10.1126/sciadv.adg0289

**Published:** 2023-06-30

**Authors:** Theinmozhi Arulraj, Hanwen Wang, Leisha A. Emens, Cesar A. Santa-Maria, Aleksander S. Popel

**Affiliations:** ^1^ Department of Biomedical Engineering, Johns Hopkins University School of Medicine, Baltimore, MD 21205, USA.; ^2^ University of Pittsburgh Medical Center, Hillman Cancer Center, Pittsburgh, PA, 15213, USA.; ^3^ Department of Oncology, and the Sidney Kimmel Comprehensive Cancer Center, Johns Hopkins University School of Medicine, Baltimore, MD 21205, USA.

## Abstract

Triple-negative breast cancer (TNBC), a highly metastatic breast cancer subtype, has limited treatment options. While a small number of patients attain clinical benefit with single-agent checkpoint inhibitors, identifying these patients before the therapy remains challenging. Here, we developed a transcriptome-informed quantitative systems pharmacology model of metastatic TNBC by integrating heterogenous metastatic tumors. In silico clinical trial with an anti–PD-1 drug, pembrolizumab, predicted that several features, such as the density of antigen-presenting cells, the fraction of cytotoxic T cells in lymph nodes, and the richness of cancer clones in tumors, could serve individually as biomarkers but had a higher predictive power as combinations of two biomarkers. We showed that PD-1 inhibition neither consistently enhanced all antitumorigenic factors nor suppressed all protumorigenic factors but ultimately reduced the tumor carrying capacity. Collectively, our predictions suggest several candidate biomarkers that might effectively predict the response to pembrolizumab monotherapy and potential therapeutic targets to develop treatment strategies for metastatic TNBC.

## Teaser

In-silico clinical trial facilitates selection of patients for immunotherapy targeting PD-1 in metastatic breast cancer.

## INTRODUCTION

Numerous hallmarks of cancer that facilitate the transformation of normal cells to neoplastic cells have been proposed (*1*). Metastasis, a process whereby cancer cells acquire invasive ability and spread from the site of origin to distant organs, is one of the hallmarks and accounts for 90% of cancer-related deaths (*2*, *3*). While the metastatic potential varies for different cancer types (*4*, *5*), a subtype of breast cancer called triple-negative breast cancer (TNBC) is characterized by a high tendency for metastasis. TNBC constitutes 15 to 20% of breast cancer and is considered a highly aggressive breast cancer subtype (*6*). TNBC tumors lack expression of estrogen and progesterone receptors, do not overexpress human epidermal growth factor receptor-2 (HER-2), and cannot be treated with drugs that target these pathways in other subtypes of breast cancer. Historically, in terms of systemic therapy, this subtype of breast cancer has relied mostly on chemotherapy in both the early-stage and metastatic settings.

TNBC tumors are usually sensitive to chemotherapy, but many patients will still relapse after treatment and median overall survival is poor (*7*–*9*). For TNBC, the first distant site of metastatic appearance is the lung (40%), followed by brain (30%), liver (20%), and bone (10%) (*10*). Genomic analysis of metastatic TNBC tumor samples from a rapid autopsy patient suggests that lung metastases promote subclonal diversification that gives rise to highly aggressive cancer clones, thus acting as “subclone incubators” (*11*).

Reduced immune cell abundance and tumor-infiltrating lymphocytes (TILs) were observed in metastatic tumors compared to primary tumors in breast cancer (*12*–*14*). Discordant programmed cell death-ligand 1 (PD-L1) expression status (*12*, *15*) and genomic alterations (*16*) between metastatic and primary tumors have also been observed. Multiple metastatic tumors in different organs or within the same organ also show substantial heterogeneity in clonal composition (*11*, *17*). Lower immunogenicity and intraindividual heterogeneity of metastatic tumors exacerbate the challenges of treating metastatic TNBC.

One of the emerging promising treatment strategies aims to enhance antitumor immune responses by targeting immune checkpoint molecules, such as cytotoxic T lymphocyte-associated antigen 4 (CTLA-4), programmed cell death protein 1 (PD-1), and PD-L1 (*18*, *19*). Pembrolizumab, a PD-1 inhibitor, boosts T cell responses by blocking the interactions of PD-1 receptor with its ligands PD-L1 and PD-L2 and has been tested in a number of clinical trials of early-stage and metastatic TNBC (*20*–*25*). When pembrolizumab was combined with chemotherapy in the KEYNOTE-355 trial, progression-free and overall survival were improved in patients with tumors with a PD-L1 combined positive score (CPS) of 10 or greater (*25*, *26*), subsequently leading to its approval in the treatment of metastatic PD-L1 CPS ≥ 10 TNBC.

When tested as a single agent in patients with metastatic TNBC, response rates were in the range of 5 to 21% depending on the tumor PD-L1 status and prior treatments received by patients (*20*–*23*). In randomized trials, pembrolizumab monotherapy has shown low response rates, and overall survival was not significantly enhanced compared to chemotherapy (*22*).

With pembrolizumab monotherapy, objective response rates were lower in previously treated patients compared to untreated patients (*20*, *23*), and a subset of patients demonstrated complete and durable response (*22*). Furthermore, with increasing levels of PD-L1 expression in the tumor, a trend toward increasing response rates and overall survival was observed (*22*, *27*). These findings highlight the need to identify predictive biomarkers for clinical benefit from pembrolizumab monotherapy. An extensive analysis of biomarkers in clinical studies presents multiple technical challenges that could be circumvented by exploratory analysis in silico followed by validation in clinical studies.

Quantitative systems pharmacology (QSP) models integrate detailed mechanisms of biological systems with drug pharmacology and assist in drug discovery by promoting a system level understanding of treatment responses (*28*). QSP models have been developed to investigate the action of various immune checkpoint inhibitors and T cell engagers (*29*, *30*), in cancer types such as colorectal cancer (*31*, *32*), breast cancer (*33*–*36*), prostate cancer (*37*), non–small cell lung cancer (*38*), and melanoma (*39*). Such models have been used in investigating treatment responses as well as dose optimization and scheduling, have been used in identifying and evaluating biomarkers and preclinical to clinical translation, and were able to explain clinically observed interindividual heterogeneity in treatment responses. As intraindividual heterogeneity is also critical in determining treatment responses in the metastatic setting, Kumar *et al.* (*40*) developed a QSP model of metastatic melanoma where multiple heterogenous tumors were explicitly represented, parameterized the model using clinical data from ipilimumab and pembrolizumab monotherapies, and then predicted the response to combination therapy. Model predictions suggested that only tumors with intermediate levels of TILs, referred to as “warm tumors,” showed a substantial enhancement in response to combination therapy compared to monotherapies (*40*).

Because of the high incidence of metastasis in TNBC, understanding the role of intraindividual heterogeneity in treatment responses is critical. In this study, we developed a transcriptome-informed QSP model with a special focus on lung metastases to investigate patient responses to pembrolizumab monotherapy as a second- or third-line therapy in metastatic TNBC. By performing virtual clinical trials, we characterized the effects of pembrolizumab on the tumor microenvironment in responders and nonresponders and predicted biomarkers potentially associated with clinical benefit.

## RESULTS

### QSP model development and calibration

**Fig. 1.**
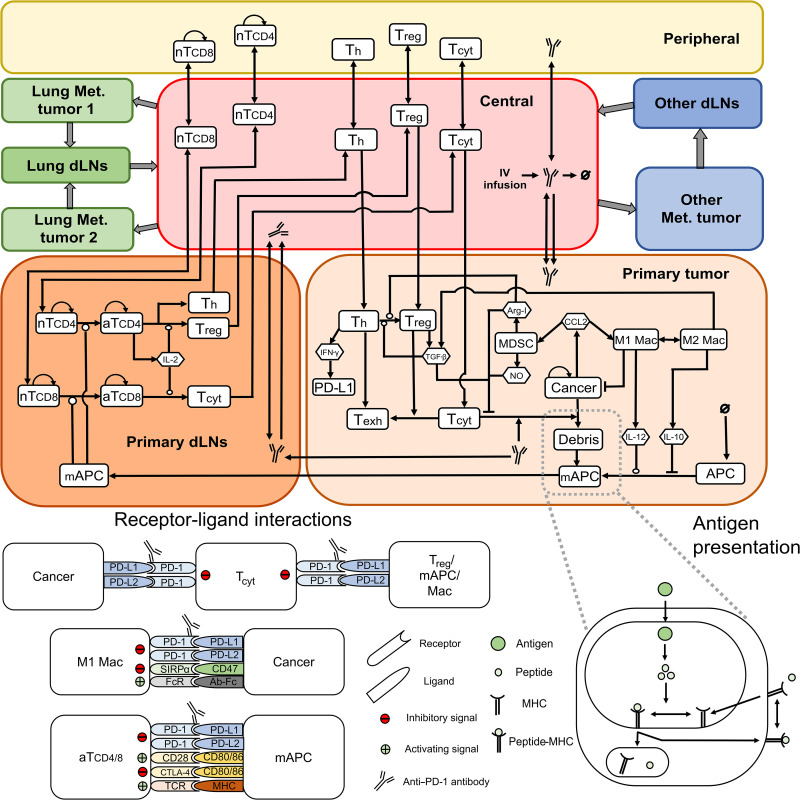
Schematic representation of the QSP model with metastatic tumors. The model includes nine main compartments: central, peripheral, and primary tumor; two lung metastatic tumors; other metastatic tumor; and three tumor-draining lymph node (each lymph node compartment associated with primary, lung, or other metastatic tumors) compartments. Mechanisms considered include interactions of cancer cells with immune cells in tumor compartments, activation of T cells in lymph node compartments, and T cell trafficking between compartments. Details of checkpoint interactions and antigen processing module are shown at the bottom of the figure. Mechanisms considered in the metastatic tumors and associated draining lymph node compartments are the same as mechanisms shown in the primary tumor and associated lymph node compartments, respectively. Th, helper T cell; T_reg_, regulatory T cell; T_cyt_, cytotoxic T cell; nTCD4/8, naïve CD4/CD8 T cell; aTCD4/8, activated CD4/8 T cell; IV, intravenous; APC, antigen-presenting cell; mAPC, mature antigen-presenting cell; Mac, macrophage; MDSC, myeloid-derived suppressor cell; Arg-I, arginase I; NO, nitric oxide; MHC, major histocompatibility complex; dLNs, draining lymph nodes.

**Fig. 2.**
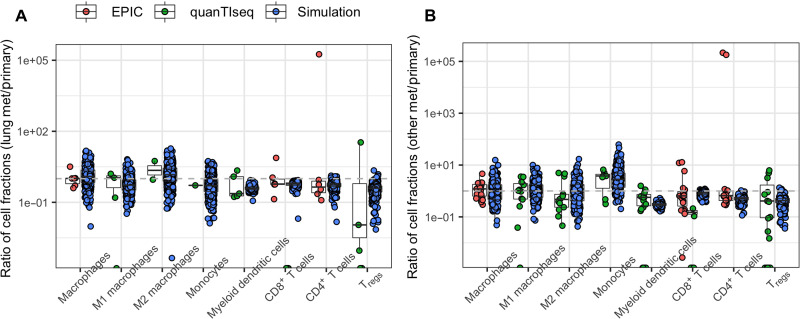
Model calibration. (**A** and **B**) Comparison of cell fractions estimated from transcriptomic data by Siegel *et al.* (*71*) using EPIC and quanTIseq with cell fractions in simulated virtual patients at baseline for lung metastatic tumors (A) and other metastatic tumors (B). Cell fractions are calculated as the count of individual cell types divided by the total number of cells in the tumor including cancer and immune cells. Values are expressed as ratio of cell fraction in metastatic tumors and matched primary tumors. Abundance of MDSCs in the QSP model was calibrated using abundance estimates of monocytes from transcriptomic data.

**Fig. 3.**
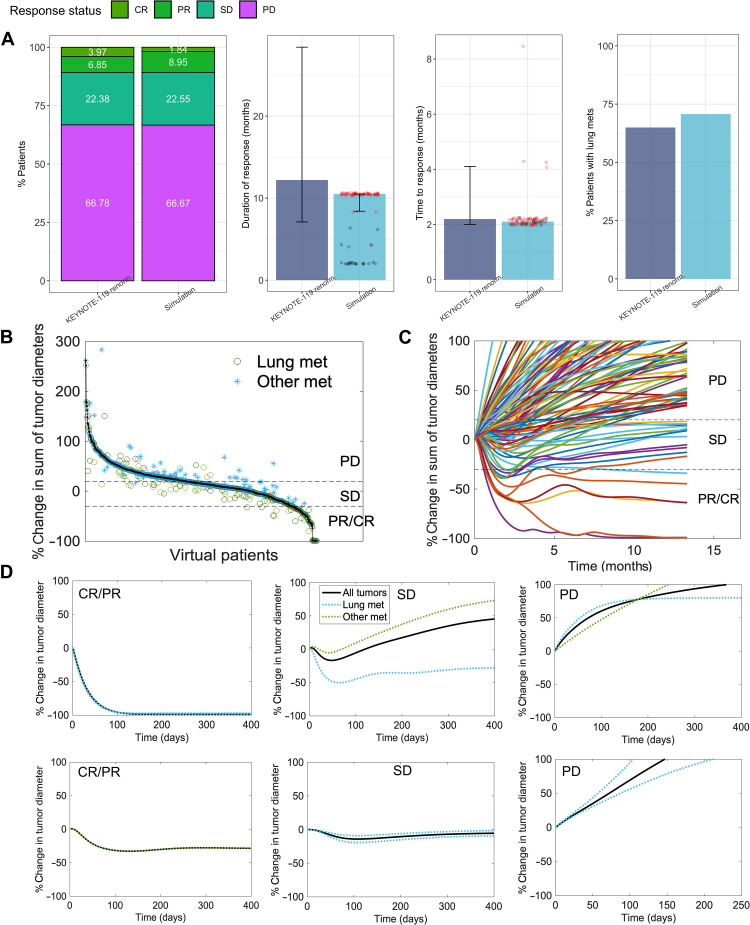
Virtual clinical trials of pembrolizumab monotherapy in metastatic TNBC. (**A**) Response rates, duration of response, time to response, and percentage of patients with lung metastases in the virtual clinical trial compared to KEYNOTE-119 trial. Data from (*22*, *27*) were renormalized by excluding nonevaluable patients. Duration of response and time to response of individual patients are shown as dots. Red dots indicate patients who did not show disease progression until the end of the simulation. (**B**) Variant of waterfall plot showing the percentage change in the sum of tumor diameter of all simulated lesions (black dots) in individual virtual patients together with the response of individual tumors (green circles and blue stars for lung and other metastatic tumors, respectively). Horizontal axis represents individual patients. (**C**) Spider plot showing time series of percentage change in sum of tumor diameter for 100 randomly sampled virtual patients. (**D**) Percentage change in individual metastatic tumor diameters of representative virtual patients with different response status based on RECIST v1.1. CR/PR, complete response/partial response; SD, stable disease; PD, Progressive disease.

We developed a QSP model with metastatic tumors to explore the effects of pembrolizumab monotherapy in metastatic TNBC (Fig. 1). This QSP model uses components of a previously developed QSP model with single TNBC tumor and was extended in this study to incorporate multiple metastatic tumors. Because lung metastases are frequently observed in patients with TNBC and have a unique tumor microenvironment (figs. S1 and S2), we explicitly incorporated lung metastatic tumors in the QSP model by adding two lung tumor compartments that drain into a lung-draining lymph node compartment. In addition, we also incorporated an “other” tumor compartment representing any metastatic tumor other than the lung and an associated other tumor-draining lymph node compartment (Fig. 1). We calibrated the relative abundance of immune cells in the lung metastatic tumors (with respect to matched primary tumors) in the QSP model using transcriptome-derived relative abundance estimates from lung metastases (Fig. 2A). Only the abundance estimates of EPIC and quanTIseq were used for model calibration because the estimates are expressed as cell fractions and enable direct comparison with cell fractions from simulations. Similarly, we used the transcriptome-derived relative abundance of cell types in metastatic tumors, excluding the lung, brain, and lymph node samples, to calibrate the immune cell abundance of other metastatic tumors in the QSP model (Fig. 2B). As the availability of transcriptomic data was limited to a low number of tumor samples, the estimates obtained using EPIC and quanTIseq were only treated as rough estimates, and an exact quantitative comparison with simulation results was not performed. We further calibrated the QSP model using data from the KEYNOTE-119 clinical trial (*22*, *27*). We generated 1000 virtual patients by selectively varying parameters of the QSP model. Among the 1000 virtual patients, 816 virtual patients who met the target tumor diameter criteria were selected for treatment simulations and subsequent analyses. We assumed that most patients underwent surgery to remove the primary tumor and incorporated this assumption in the model by setting the initial number of cancer cells in the primary tumor compartment to 0. Metastatic tumor compartments were seeded at randomly sampled time points until at least one metastatic tumor reached the target tumor diameter, resulting in a varying number of established metastatic tumors in virtual patients with a maximum of two lung metastatic tumors and one other metastatic tumor. We simulated the action of pembrolizumab monotherapy in virtual patients by administering 200 mg of pembrolizumab every 3 weeks and calibrated the QSP model parameters to recapitulate clinically observed response rates, duration of response, time to response, and the percentage of patients with lung metastases in the virtual clinical trial (Fig. 3A). While a subset of responders showed cancer progression during the course of the treatment, there were also virtual patients who did not progress until the end of the simulation (Fig. 3A, red dots), mimicking patients with long-term response in the clinical trial. As expected, a large intraindividual and interindividual heterogeneity in treatment response was observed among virtual patients (Fig. 3, B to D).

### Impact of pembrolizumab treatment on the tumor microenvironment

It is well-known that pembrolizumab enhances the effector function of T cells by blocking the interaction of the PD-1 receptor with its ligands PD-L1 and PD-L2, but the implications of this blockade on the composition and properties of tumor microenvironment are not clear. To characterize this, we compared simulations of virtual patients in the presence and absence of pembrolizumab therapy (fig. S3). First, we observed an increase in the density of T cells in the central compartment (fig. S3A), which was expected because of the increased activation of T cells induced by an increase in the cancer cell killing upon the action of pembrolizumab. In the central compartment, an increase in the diversity of cytotoxic T cells and an increase in the ratio of regulatory T cells (T_regs_) and cytotoxic T cells were also observed in pembrolizumab-treated virtual patients (fig. S3, B and C).

Upon pembrolizumab treatment, there was a notable increase in the fraction of immune cells in metastatic tumors including both protumorigenic and antitumorigenic immune cells (fig. S3E). This increase was accompanied by a reduction in the ratio of protumorigenic and antitumorigenic cell types, indicating an increased antitumor activity (fig. S3F). However, individual cell types showed different trends. The density of tumor-infiltrating cytotoxic T cells and T_regs_ was increased upon pembrolizumab treatment (fig. S3, H and I). Unlike in the central compartment, a transient decrease in the diversity of cytotoxic T cells and a decrease in the ratio of Tregs and cytotoxic T cells were observed in the tumor (fig. S3, G and J).

A transient increase in the density of macrophages and myeloid-derived suppressor cells (MDSCs) was also observed in the tumor (fig. S3, K and M). Unexpectedly, the ratio of M2 to M1 macrophages was higher upon pembrolizumab treatment (fig. S3L). These findings suggest that the action of pembrolizumab does not consistently enhance all antitumorigenic factors or suppresses all protumorigenic factors. Pembrolizumab treatment also altered the predicted levels of cytokines (fig. S4). Concentrations of interferon-γ (IFN-γ), transforming growth factor–β (TGF-β), and interleukin-2 (IL-2) were increased, but concentrations of C-C motif chemokine ligand 2 (CCL2), IL-10, and IL-12 were decreased. Anti–PD-1 treatment increased the expression of PD-L1 in the tumor (fig. S3O), decreased the secretion of angiogenic factors (fig. S4B), and reduced the carrying capacity of the metastatic tumors (fig. S4H). Ultimately, richness of the cancer clones in the tumor was also decreased, suggesting the elimination of pembrolizumab-sensitive cancer clones (fig. S3D).

**Fig. 4.**
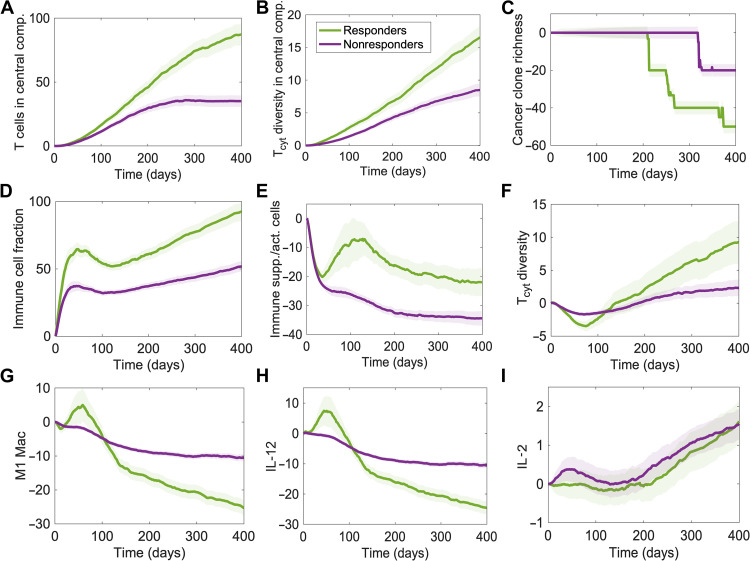
Effect of pembrolizumab monotherapy on model species in responders and nonresponders. Time series of percent change in the (**A**) density of activated T cells in the central compartment, (**B**) diversity of cytotoxic T cells in the central compartment, (**C**) richness of cancer clones in tumors, (**D**) fraction of immune cells in the tumor, (**E**) ratio of the densities of immune-suppressive and immune-activating cell types in the tumor, (**F**) diversity of cytotoxic T cells in the tumor, (**G**) density of M1 macrophages in the tumor, (**H**) concentration of IL-12 in the tumor, and (**I**) concentration of IL-2 in lymph node compartments, in treatment simulations of virtual patients with respect to simulations of same virtual patient cohort without any treatment. Quantities from tumor and lymph node compartments represent average values from all metastatic tumors simulated and lymph node compartments, respectively. Solid curves show median values of virtual patients in each group. Shaded regions represent 95% confidence intervals. Colors represent response status. Patients with CR/PR and SD were considered as responders.

We then compared the effects of pembrolizumab on immune cell composition and cytokine levels between responders and nonresponders by stratifying virtual patients based on the response status (Fig. 4 and fig. S5). In general, the aforementioned effects on cellular and molecular species observed in the entire cohort of virtual patients were quantitatively enhanced in responders compared to nonresponders. Increases in the density and diversity of T cells in the central compartment and the fraction of immune cells in the tumor were higher in responders compared to nonresponders (Fig. 4, A, B, and D). Similarly, the decrease in the richness of cancer clones was greater in responders, suggesting increased clonal extinction (Fig. 4C). A transient decrease in the tumor-infiltrating cytotoxic T cell diversity was also greater in responders (Fig. 4F). Apart from these prominent quantitative differences, mild qualitative differences were observed between responders and nonresponders. While the ratio of protumorigenic and antitumorigenic immune cell densities consistently decreased in nonresponders, a transient increase was observed in responders following an initial decrease (Fig. 4E). Similarly, a transient increase in the density of M1 macrophages and IL-12 levels was seen in responders but was not observed in nonresponders (Fig. 4, G and H). Although IL-2 levels were increased upon pembrolizumab therapy in both responders and nonresponders, the increase tended to be earlier in nonresponders (Fig. 4I).

### Identification and ranking of potential biomarkers

**Fig. 5.**
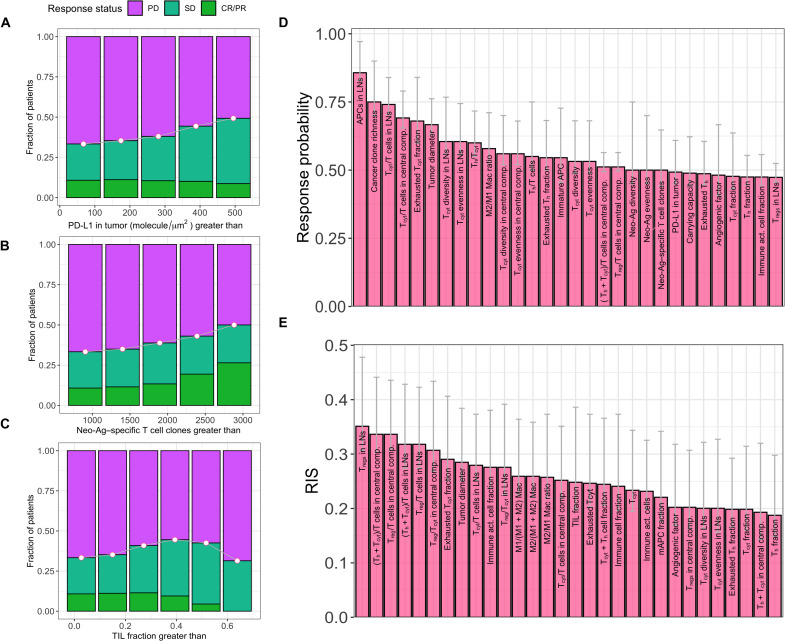
Model predictions of individual biomarkers. (**A** to **C**) Response status of virtual patients in subgroups generated on the basis of increasing threshold levels of PD-L1 expression in the tumor (A), number of neo-antigen–specific T cell clones (B), and fraction of tumor-infiltrating lymphocytes (C). (**D** and **E**) Best 30 biomarkers identified on the basis of response probability (D) or responder inclusion score (RIS) (E). Response probability is calculated as the fraction of responders among all patients in a virtual patient subgroup. RIS is calculated as the difference between fraction of responders and nonresponders present in a subgroup among all responders or nonresponders in the entire patient cohort (see Eq. 1). Error bars represent 95% confidence intervals calculated by bootstrapping. Patients with CR/PR and SD were considered as responders. Ag, antigen; TIL, tumor-infiltrating lymphocyte; APC, antigen-presenting cell (mature and immature); LN, lymph node.

We then sought to identify biomarkers that could potentially identify responders to pembrolizumab monotherapy in metastatic TNBC. First, we tested whether the baseline levels of previously identified biomarkers such as tumor PD-L1 expression, the number of neo-antigen–specific T cell clones, and the fraction of TILs correlated with response to pembrolizumab monotherapy. In this analysis, neo-antigen–specific T cell clones were considered as a proxy to tumor mutational burden due to the correlation between these two quantities (*41*). On the basis of the levels of each of the three biomarkers individually, we generated virtual patient subgroups (see Materials and Methods). We then calculated the fraction of responders among all the patients in each subgroup, hereafter termed as response probability. As expected, we observed a modest increase in response probability among virtual patient subgroups as the threshold levels of these biomarkers were increased (Fig. 5, A to C). Correlation between the PD-L1 expression and fraction of TILs was predicted to be 0.56 (fig. S6), similar to the clinically reported correlation of 0.45 (*42*).

We performed an exhaustive testing of biomarker candidates (see Materials and Methods) in the QSP model to search for other biomarkers with a potentially higher predictive power. For each biomarker candidate, we generated virtual patient subgroups and calculated the response probability for each subgroup as previously described. We then ranked the biomarker candidates based on the highest response probability achieved among the virtual patient subgroups generated for each biomarker candidate.

Among the biomarker candidates tested, the density of antigen-presenting cells (APCs) in tumor-draining lymph nodes achieved the highest response probability of 0.86 (Fig. 5D). This was followed by the richness of cancer clones, the fraction of cytotoxic T cells among all T cells in lymph node and central compartments, the fraction of exhausted cytotoxic T cells in metastatic tumors, the tumor diameter, and the diversity and evenness of cytotoxic T cells in lymph nodes. Each of these biomarker candidates achieved a response probability higher than 0.6. Two biomarkers with known clinical relevance, such as tumor PD-L1 expression and the number of neo-antigen–specific T cell clones, were among the best 30 biomarker candidates identified, as both achieved a response probability of approximately 0.5. The fraction of TILs achieved a response probability of 0.45, suggesting that it is a slightly weaker biomarker compared to PD-L1 expression in the tumor.

We tested whether the virtual patient subgroups selected based on identified biomarkers incorporated a considerable number of responders from the whole virtual patient cohort. The virtual patient subgroup chosen based on the density of APCs in lymph nodes, which is the best biomarker identified, included only 11% of responders from the entire virtual patient cohort despite achieving a remarkable response probability (fraction of responders within a selected patient subset rather than the entire patient cohort) of 0.86 (fig. S7A). In other words, while this biomarker effectively identifies a small subset of responders from the whole cohort (11%), the majority of responders from the whole cohort (89%) are ignored and are not benefitted by this biomarker. Among the virtual patient subgroups chosen based on best 30 biomarkers identified, three biomarkers—the density of T_regs_ in lymph nodes, the fraction of helper and cytotoxic T cells in the central compartment, and the fraction of T_regs_ in the central compartment—also had higher percentage of responders from the whole cohort (fig. S7A).

To maximize the number of responders in the best subgroups selected, we introduced a metric that we call responder inclusion score (RIS). RIS is calculated as the difference between the fraction of responders and the fraction of nonresponders from the entire cohort present in the subgroup. The first term maximizes the number of responders while the second term minimizes the number of nonresponders in the subgroup with respect to the total number of responders or nonresponders in the entire cohort. When biomarker candidates were ranked on the basis of the RIS rather than response probability, the density of T_regs_ in lymph nodes was the best biomarker with a highest RIS of 0.35 (Fig. 5E). The virtual patient subgroup identified based on this biomarker incorporated 79% of responders and 43% of nonresponders from the entire cohort (fig. S7B). The best 30 biomarkers chosen based on RIS included the fraction of TILs that attained a RIS of 0.24. The fraction of TILs is predicted to be a better biomarker in maximizing RIS compared to PD-L1 in tumor and neo-antigen–specific T cell clones, although the latter biomarkers achieved higher response probabilities. Several biomarkers identified as the best 30 based on response probability were also among the best 30 when ranked on the basis of the RIS (biomarkers highlighted in data S2). This includes biomarkers such as the fraction of cytotoxic T cells in lymph nodes/central compartment, fraction of exhausted cytotoxic T cells in the tumor, and the initial tumor diameter. However, the threshold biomarker levels for the virtual patient subgroup that attains the highest response probability or RIS varied (data S2). Together, individual biomarkers tested were able to achieve a maximum RIS of 0.35 and a maximum response probability of 0.86.

### Predictive power of combinations of two biomarkers

**Fig. 6.**
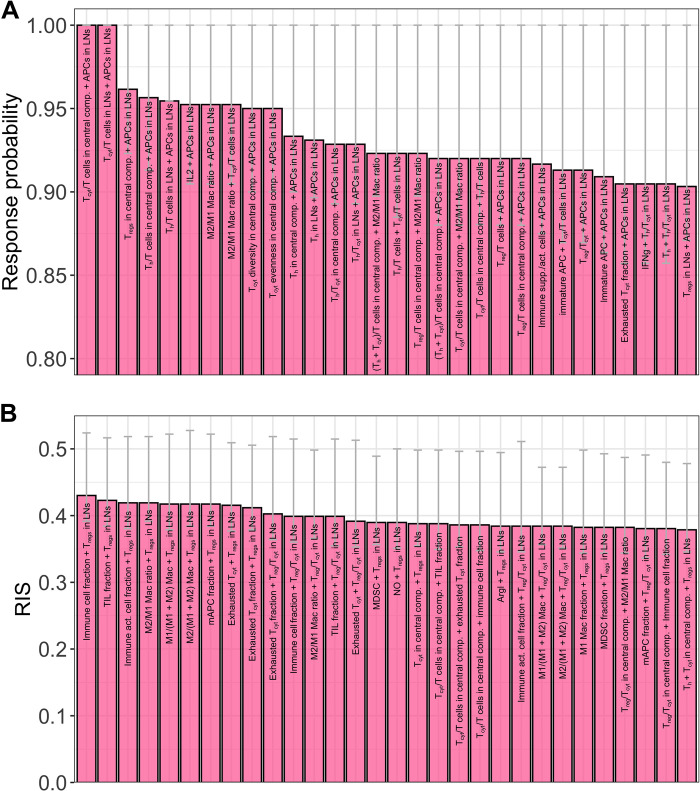
Model predictions of biomarker combinations. Best 30 combinations of two biomarkers identified based on response probability (**A**) or RIS (**B**). Response probability is calculated as the fraction of responders among all patients in a virtual patient subgroup. RIS is calculated as the difference between fraction of responders and nonresponders present in a subgroup among all responders or nonresponders in the entire patient cohort (see Eq. 1). Error bars represent 95% confidence intervals calculated by bootstrapping. IFNg, interferon-γ.

As the predictive power of individual biomarkers could be limited, we tested whether any combination of two biomarkers achieved higher predictive power. We generated all possible combinations of two biomarker candidates and generated subgroups of virtual patients on the basis of the levels of both biomarker candidates in the combination. The combination of the fraction of cytotoxic T cells in the central compartment with the density of APCs in lymph node achieved a response probability of 1 (Fig. 6A). Similarly, the fraction of cytotoxic T cells in lymph nodes combined with the density of APCs in lymph node also attained a response probability of 1 (Fig. 6A). When the biomarker threshold levels for virtual subgroups were chosen to maximize response probability, the fraction of responders from the entire cohort included in the subgroups were low as observed for single biomarkers (fig. S8A).

When biomarker combinations were ranked on the basis of the RIS, the highest RIS attained was 0.43 for the combination of immune cell fraction in metastatic tumors and the density of T_regs_ in lymph nodes (Fig. 6B). On the basis of the levels of these biomarker combinations, 72% of responders in the overall cohort were included in the patient subgroup, but only 29% of nonresponders were included (fig. S8B). For all the biomarker combinations, we calculated the percentage change in RIS/response probability in combination compared to the best individual biomarker in that combination. The increase in predictive power varied greatly among pairs of biomarkers but was considerably enhanced compared to individual ones, suggesting a higher predictive power of biomarker combinations (fig. S9). Despite only small differences in the response probability/RIS attained among top-ranked biomarkers, the differences were statistically significant (figs. S10 and S11).

### Dependence of response rate on model parameters

**Fig. 7.**
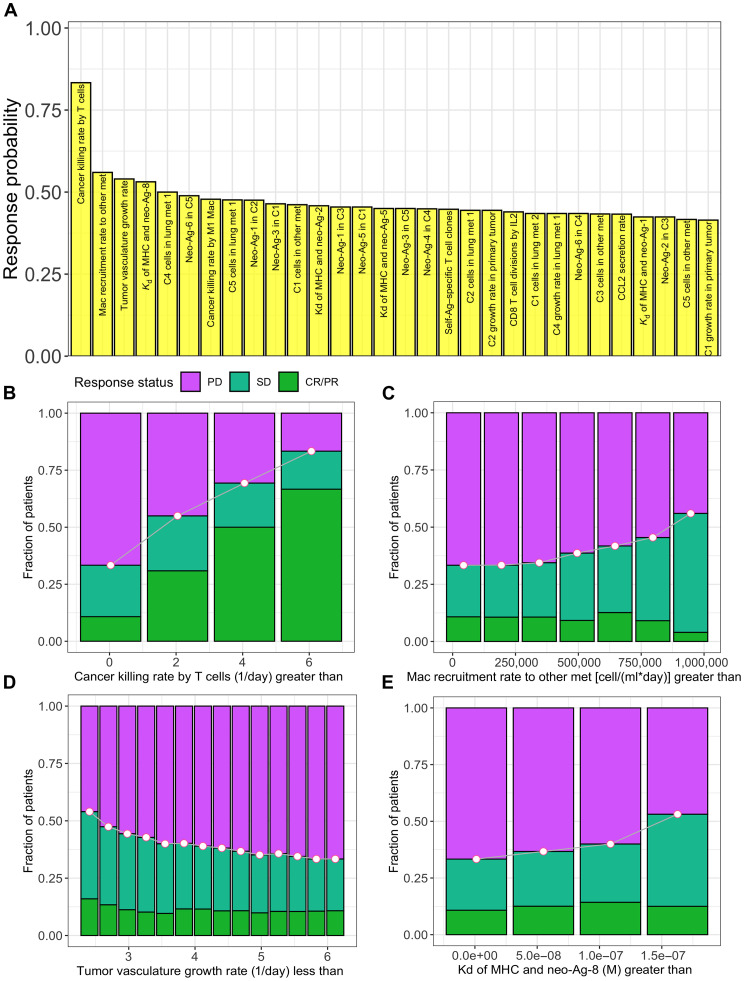
Dependence of response rates on model parameters. (**A**) Most influential model parameters. (**B** to **E**) Response status in virtual patient subgroups identified on the basis of parameter values: killing rate of cancer cells by cytotoxic T cells (B), macrophage recruitment rate to other metastatic tumor (C), tumor vasculature growth rate (D), and dissociation constant of MHC and peptide from neo-antigen 8 (E). Response probability is calculated as the fraction of responders among all patients in a virtual patient subgroup. C*i*, *i*th cancer clone; *K*_d_, dissociation constant. For each parameter, 15 virtual patient subgroups were considered, and subgroups having less than 20 patients were eliminated.

To identify the most influential parameters in the QSP model, we chose multiple cutoffs for each parameter in the allowable range and generated virtual patient subgroups with parameter values above and below chosen cutoffs. Similar to the biomarker analysis discussed in the previous section, parameters were ranked on the basis of the maximum response probability attained in the subgroups generated. Maximum response probability attained can be considered as a measure of how much the response probability is increased by a particular parameter in a patient subset compared to the entire patient cohort. Therefore, parameters attaining highest response probability were considered as the most influential in determining response rates. Killing rate constant of cancer cells by cytotoxic T cells was the most influential parameter followed by parameters such as macrophage recruitment rate to the other metastatic tumor, tumor vasculature growth rate, and dissociation constant of peptide–major histocompatibility complex (MHC) (Fig. 7A). While increasing the threshold levels for parameters such as cancer cell killing rate, the macrophage recruitment rate resulted in increased fraction of responders; the tumor vasculature growth rate showed the opposite trend (Fig. 7, B to E).

We chose 18 model parameters that were fixed among virtual patients and tested the influence on the overall response rates by perturbing the parameter values (50% decrease to 50% increase) (fig. S12). Among the tested parameters, overall response rates were sensitive to parameters including half-maximal CCL2 level for MDSC recruitment, T cell diversity, rate constant for the degradation of CCL2, rate constant of dead cell clearance from the tumor compartments, rate constant of T cell death by T_regs_, and the concentration of self-antigen in cancer cells. Response statuses of a notable fraction of patients were shifted from progressive disease (PD) to stable disease (SD) or partial response (PR) by increasing parameters such as T cell diversity, half-maximal CCL2 level for MDSC recruitment, and rate constant for the degradation of CCL2 (fig. S13). This suggests that a lower T cell diversity or a higher concentration of CCL2 that induces the recruitment of MDSCs and macrophages could be an important factor impeding patient responses. On the other hand, a reverse trend with predominant shift from SD/PR to PD was observed by increasing the values of parameters such as the rate constant of T cell death by T_regs_ and the concentration of self-antigen in cancer cells.

### Heterogeneity in the response of metastatic tumors to pembrolizumab therapy

**Fig. 8.**
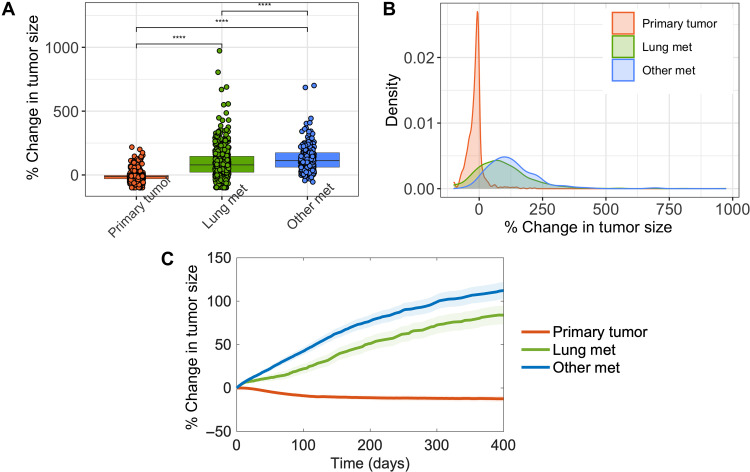
Comparison of primary and metastatic tumor response to pembrolizumab monotherapy. (**A**) Box plot showing the percent change in individual tumor diameters at the end of treatment simulation with respect to the baseline tumor diameter. (**B**) Density plot of the treatment response of primary and metastatic tumors. (**C**) Time series of primary and metastatic tumor response after the start of pembrolizumab treatment. Colors represent tumors from different organs. Statistical significance was computed by Wilcoxon test (****P < 0.0001).

Next, we considered a different cohort in which virtual patients are assumed to have intact primary tumor in addition to metastatic tumors. We sought to explore whether the differences in immune microenvironment of primary and metastatic tumors assumed in this study are sufficient to confer differences in response to pembrolizumab therapy. We simulated the response of virtual patients to pembrolizumab monotherapy by administering 200 mg of pembrolizumab every 3 weeks. We compared the increase in primary and metastatic tumor load 400 days after the start of pembrolizumab treatment (Fig. 8). The median primary tumor response to pembrolizumab was higher compared to lung and other metastatic tumors (Fig. 8, A and C). Lung metastatic tumors also showed a significantly better response compared to other metastatic tumors (Fig. 8, A and C). We also observed a large heterogeneity in the response of primary and metastatic tumors (Fig. 8B). Notably, the heterogeneity was higher in both lung and other metastatic tumors compared to primary tumors (Fig. 8B).

## DISCUSSION

The treatment of TNBC is challenging owing to its high propensity of metastatic spread and the establishment of metastatic tumors at distant sites such as the lung, liver, brain, and bones (*7*, *8*, *10*). The challenge of developing effective therapeutic strategies is further exacerbated by the intraindividual heterogeneity of metastatic tumors that ultimately leads to heterogeneity in treatment responses. Pembrolizumab monotherapy has demonstrated durable responses in small numbers of patients with previously treated metastatic TNBC (*22*). However, biomarkers that reliably identify the subgroup of patients who are most likely to achieve therapeutic benefit are now lacking.

In this study, we developed a QSP model for metastatic TNBC that incorporates multiple metastatic tumors to address these challenges in investigating the effects of pembrolizumab. Because lung metastases are observed in most patients with TNBC, we explicitly incorporated metastatic lung tumors into the model. Typically, QSP model development and parameterization rely on data from clinical, preclinical, and in vitro studies. Given the sparsity of such data for metastatic tumors from specific sites, we used transcriptomic data as suggested previously by Lazarou *et al.* (*43*) to calibrate the differences in immune cell content of the lung and other metastatic tumor site relative to the primary breast tumor. A virtual clinical trial simulation with the calibrated model was able to capture heterogeneity in the response of tumors within individual patients.

Simulations predicted that pembrolizumab monotherapy not only increased the tumor infiltration of immune-activating cells but also increased immune-suppressive cell types and cytokines. These predictions suggest that a combination of pembrolizumab with therapies targeting immune-suppressive cell types/cytokines such as M2 macrophages, MDSCs, TGF-β, or IL-10 could potentially be beneficial in further increasing overall response rates. We observed that pembrolizumab treatment had similar qualitative effects on most cell types and molecular species in responders and nonresponders except M1 macrophages and IL-12 levels.

Higher levels of biomarkers such as PD-L1 expression, TILs, and tumor mutational burden have been associated with better response (*44*). We observed an increase in response rates, when patient subgroups with increasing thresholds of PD-L1 expression were analyzed, a trend that was also seen in the KEYNOTE-119 trial (*22*, *27*). While PD-L1 expression is a commonly used biomarker in metastatic TNBC, there is a need for better biomarkers because a substantial fraction of patients with high PD-L1 expression are nonresponders and some patients with low PD-L1 expression may respond to pembrolizumab monotherapy (*45*). We predicted several biomarkers that might potentially confer higher predictive power, such as the richness of cancer clones, the fraction of cytotoxic T cells in lymph node and blood, the fraction of exhausted cytotoxic T cells in metastatic tumors, and the diversity and evenness of cytotoxic T cells in lymph nodes. These predictions suggest that, when virtual patients are selected on the basis of the identified biomarkers with an appropriate threshold biomarker level, the highest response probability that could be achieved in the patient subgroup is 0.86. Because of limited data availability on biomarker analysis, a rigorous quantitative validation of the model could not be performed, and our work generates hypotheses that need to be clinically validated. The proposed predictive biomarkers include those from biopsies and blood samples and can be measured; while biomarkers such as the densities of immune cells can be measured by flow cytometry with appropriate markers, biomarkers such as the richness of cancer clones and the diversity or evenness of cytotoxic T cells would require genomic and T cell receptor (TCR) sequencing. The presence of predictive biomarkers from blood samples among top-rated biomarkers suggests the feasibility of testing biomarkers in cases where obtaining biopsies might be difficult depending on the site of metastases.

We found that biomarkers that resulted in higher response rates in a chosen subset of patients did not necessarily include a large proportion of responders from the population. In addition, only a small fraction of responders from the entire cohort were selected when biomarkers identified to solely maximize response probability were used to select virtual patient subgroups. We thus introduced a metric called RIS to rank biomarkers based on their ability to stratify the responders and nonresponders in the entire patient cohort. While the number of responders was higher in virtual patient subsets selected based on RIS, even a combination of two biomarkers was not able to effectively eliminate the inclusion of nonresponders. Considering the complexity of the tumor microenvironment, we propose that the combination of more than two biomarkers might be required to effectively stratify responders and nonresponders and that future studies focusing on identifying the minimal set of biomarkers required would be invaluable.

Parameters of the QSP model presented were estimated using available in vitro, in vivo, and clinical data. While 12% of the parameters do not have strong experimental support, these parameters were extensively calibrated to fit the clinical response data. However, considering the differences in experimental conditions and tumor samples used, estimated parameters should only be treated as rough estimates that could be refined upon the availability of more data. Sensitivity analysis has identified parameters that, when perturbed, could shift the response status of a substantial fraction of virtual patients. Thus, note that the predictions of the model could rely on parameter values and that model predictions need confirmation from clinical studies. Availability of more data for model calibration and validation would further solidify the ability of the model to generate prospective predictions. Among the model parameters, a subset (127 parameters) was chosen to vary between virtual patients to account for the heterogeneity in patient responses and the uncertainty in parameter values. Compared to previous studies (*33*, *36*), a greater number of parameters were varied in this study to account for tumor-tumor heterogeneity within a patient, in addition to interindividual variability. This includes model parameters known to be highly variable among patients such as the initial tumor diameter, PD-L1 expression, and tumor growth rate constant. Parameter distributions for sampling these parameters were estimated by fitting the clinical response data to ensure that there is adequate variability in virtual patient responses. The choice of parameters to vary and their ranges/distributions need to be fine-tuned upon the availability of patient-level data to ensure that the virtual patients generated are realistic.

In this study, we focused on predicting the responses in patients with previously treated metastatic TNBC. The combination of chemotherapy with pembrolizumab was tested in the first-line setting and is approved for the treatment of patients with metastatic PD-L1^+^ TNBC (*25*, *26*). The model and workflow presented in this study could be used to simulate the combination of chemotherapy with pembrolizumab upon the addition of modules for pharmacokinetics and pharmacodynamics of chemotherapies and recalibration of the model parameters to represent the first-line setting. Considering the higher incidence of adverse effects when chemotherapy is combined with pembrolizumab, careful selection of patients for either pembrolizumab monotherapy or pembrolizumab in combination with chemotherapy would also be beneficial in the first-line setting. Similarly, complete disappearance of metastatic tumors was observed in a subset of patients with TNBC treated with atezolizumab alone (*46*, *47*) or in combination with nab-paclitaxel (*48*), suggesting that the identification of appropriate biomarkers would be highly beneficial for patients treated with these drugs (where available) as well. Thus, a comparative in silico analysis of chemo-immunotherapy strategies in the same virtual patient cohort would suggest whether monotherapy with pembrolizumab or atezolizumab could be beneficial for individual patients, or whether they would derive clinical benefit only if chemotherapy was combined with immunotherapy.

A previous study (*49*) demonstrated that lung metastatic tumors have higher immunogenicity compared to metastatic tumors in the brain, liver, and bones, suggesting that lung metastases might be ideal targets for immunotherapy strategies. However, not all metastatic tumors in the lung show high immunogenicity, suggesting a heterogeneity in treatment responses (*49*). Consistently, we observed that lung metastatic tumors had a large heterogeneity and, when compared to other metastatic tumors, had a better median response to pembrolizumab. Our simulation results also predicted a relatively resistant nature of metastatic tumors compared to primary tumors that could be a hindering factor in the successful treatment of metastatic TNBC with immunotherapy. It is well known that cold tumors might be relatively resistant to immunotherapy compared to hot tumors, and, in such cases, other treatment options such as chemo-immunotherapy are recommended (*50*, *51*).

One of the limitations of our study is that only target lesions were simulated and nontarget/new lesions were neglected in the response evaluation. In clinical trials, the appearance of new metastatic lesions or unequivocal progression of nontarget lesions is considered PD. In the QSP model, we did not consider the detailed process of metastasis and directly introduced cancer cells into tumor compartments at specified time points that were randomly sampled from distributions established using clinical data. This simplified approach minimizes the number of parameters required. However, upon availability of sufficient data to calibrate the model parameters, the detailed process of metastasis could be incorporated to enable a mechanistic understanding of the effect of pembrolizumab on the appearance of new metastatic lesions. Furthermore, explicitly incorporating metastatic tumors other than lung metastases could be of interest in the future. For instance, liver metastases have been associated with systemic suppression of the immune response and poor responses to immunotherapy (*52*, *53*). Investigating the response of liver metastatic tumors to various therapeutic strategies would be of importance in exploring treatment options for patients with liver metastases. The availability of patient-level data from clinical trials would facilitate such model advancements. Interaction between tumors due to cytotoxic T cell trafficking was observed to be minimal in the majority of patients in this study (fig. S14). The QSP model developed here can be extended to include mechanisms mediating tumor-tumor interactions for the investigation of abscopal effects seen in the context of local therapy.

Immune cell types such as cytotoxic T cells, helper T cells, T_regs_, APCs, MDSCs, and macrophages that have been relatively well studied in the TNBC microenvironment were included in the model. Apart from the cell types incorporated in the model, estimates of xCell (*54*), a gene signature–based cell composition inference tool predicted that lung metastatic tumors tend to have higher stromal cell content compared to the primary tumor (fig. S1D). Cancer-associated fibroblasts (CAFs) are a highly heterogenous, prevalent stromal cell type in the tumor microenvironment known to promote angiogenesis, modulate chemoresistance, and facilitate metastasis (*55*, *56*). A systemic analysis of stromal cell–derived cytokines in TNBC has also identified numerous potential therapeutic targets (*57*). Apart from the stromal cells, prior analysis of clinical samples has suggested the presence of tertiary lymphoid structures (TLSs) with or without active germinal centers in TNBC tumors (*15*). The presence of mature TLS with germinal centers has been associated with prognosis in several cancer types (*58*–*60*). These observations suggest that cell types such as CAFs and B cells might affect responses to immunotherapy. Subpopulations of cell types, such as T_regs_ and MDSCs, with distinct phenotypic characteristics and gene expression, have been identified and potentially have different functional capabilities (*61*, *62*). Incorporation of these cell types/subtypes in the QSP model in future work would enable testing whether these cell types could potentially act as biomarkers in response prediction to pembrolizumab or other therapies.

In this study, we assumed that primary and metastatic tumors differ mainly in immune cell composition. Despite this assumption, heterogeneity between individual tumors in characteristics other than immune cell composition was considered by randomly sampling parameter values such as clonal diversity and neo-antigen burden for every tumor in a virtual patient. Identical parameter distributions were used for primary and metastatic tumors. Comparing the differences between primary and metastatic tumors in clonal composition and neo-antigens using appropriate high-throughput data from clinical samples of patients with TNBC could help better inform the QSP model developed in this study. While interindividual variability and intraindividual heterogeneity in terms of tumor-tumor heterogeneity are accounted for in this study, our QSP model ignores intratumoral heterogeneity and assumes that tumors are well-mixed. Spatial immune architecture such as the proximity of certain cell types has been shown to be associated with disease prognosis (*63*). Agent-based models have been developed and integrated with QSP models to explore the effects of intratumoral heterogeneity on treatment responses (*64*–*66*). Nevertheless, the QSP platform developed in this study could be integrated with such spatial models or adapted for other metastatic cancers to investigate existing and emerging treatment strategies in silico.

## MATERIALS AND METHODS

### QSP model of metastatic TNBC

We developed a QSP model for metastatic TNBC using components of a previously developed QSP model with a single TNBC tumor (*33*). The extended QSP model consists of metastatic tumor compartments and associated lymph node compartments in addition to the central, peripheral, and primary tumor and primary tumor-draining lymph node compartments considered in the previous study (*33*). Metastatic tumor compartments include two lung metastatic tumor compartments and a single compartment to represent metastatic tumors other than lung metastases, referred to as other metastatic tumor compartment (Fig. 1). Multiple lung metastases were assumed because it is the most common site of metastasis. As per RECIST v1.1 (*67*), a maximum of two lesions per organ are chosen as target lesions for quantitative assessment. Therefore, we consider two lung metastatic tumor compartments in the QSP model as only target lesions are simulated in this study. Both lung metastatic tumor compartments are connected to a lung-draining lymph node compartment, and other metastatic tumor compartment is connected to an associated other lymph node compartment via lymphatic system. Each lymph node compartment in the QSP model represents the sum total of lymph nodes draining a particular organ. The number of metastatic tumor compartments is not a limitation of the model; it can be readily extended. The model consists of 641 equations and 737 parameters and is implemented using the SimBiology toolbox of MATLAB (MathWorks, Natick, MA). Here, we discuss extensions introduced in this study and provide only a brief description of the components of the base model. Detailed description of model equations describing dynamics of individual species is provided in (*33*). The full set of equations, chemical reactions, and model parameters is presented in the Supplementary Materials; the MATLAB code is available at http://dx.doi.org/10.17632/r46rk4vwdv.1 to ensure reproducibility.

As in the previous model, the QSP model with metastases incorporates multiple modules representing the dynamics of a species or a collection of associated species in the tumor microenvironment. These modules represent the dynamics of cell types such as cancer cells, APCs, macrophages, MDSCs, T_regs_, cytotoxic T cells, and helper T cells; cytokines such as IL-2, IL-10, IL-12, IFN-γ, TGF-β, and CCL2; angiogenic factors; and checkpoint molecules expressed on immune cells and cancer cells. Mechanisms involved in the dynamics of every model species in metastatic tumor compartments and associated lymph node compartments are assumed to be the same as the primary counterparts.

#### Brief description of the base model

Dynamics of naïve CD4^+^ and CD8^+^ T cells are modeled by considering the trafficking of naïve T cells between the central, peripheral, and lymph node compartments (*68*) and proliferation in the peripheral and lymph node compartments. Few cancer cells are incorporated into the primary tumor compartment at the start of the simulation, while metastatic tumor compartments are seeded at later time points. Dynamics of cancer cells is modeled by a modified Gompertzian growth pattern, and the dynamics of tumor vasculature is modeled as changes in tumor carrying capacity, which is defined as the maximum number of cancer cells that can be supported at a given time point. Cancer cells are assumed to secrete angiogenic factors that increase the tumor carrying capacity. Natural death or apoptosis of cancer cells is modeled as a first-order reaction and results in the release of tumor-associated neo-antigens and self-antigens into the tumor compartment.

Uptake of tumor-derived neo-antigens and self-antigens by APCs and subsequent maturation results in the migration of mature APCs to the tumor-draining lymph node compartment. Cytokine-mediated effects on APCs are modeled such that IL-12 facilitates the maturation of APCs and that IL-10 inhibits APC maturation. Detailed mechanisms of antigen processing and presentation including cleavage of proteins into peptides in the intracellular vesicles, binding of peptides to MHC molecules, and transport to the cell surface are modeled. Activation of naïve T cells depends on the extent of TCR ligation by peptide-MHC on APCs and is implemented as a Hill function. The number of divisions of activated T cells is approximated as a linear sum of contributions from: (i) TCR ligation, (ii) CD28 engagement that is competitively inhibited by CTLA-4, and (iii) IL-2 derived from helper T cells (*69*). A separate compartment representing synapse between T cell and APC is considered to model the interactions of CD28 and CTLA-4 with their shared ligands CD80 and CD86. Following activation and transport into the central compartment, infiltration of T_regs_, cytotoxic T cells, and helper T cells into the tumor compartment is assumed to depend on the tumor volume and volume fraction of vascular space in tumor. Tumor-infiltrating cytotoxic T cells kill cancer cells with a rate depending on the ratio of cytotoxic T cells and cancer cells and result in the enhanced release of tumor-associated antigens. Tumor-infiltrating cytotoxic T cells and helper T cells are assumed to become exhausted by the interaction of PD-1 with ligands on cancer cells and by the action of IL-10 from T_regs_.

Cancer cells secrete CCL2 that is assumed to mediate the recruitment of M1 macrophages and MDSCs into the tumor compartment. M1 macrophages undergo reversible polarization to M2 macrophages. M1 macrophages are the source of IL-12, and M2 macrophages secrete angiogenic factors, TGF-β, and IL-10. Cytokines IL-10 and TGF-β promote the polarization of M1 to M2 macrophages, whereas cytokines IL-12 and IFN-γ promote the polarization of M2 to M1 macrophages. Phagocytosis of cancer cells by M1 macrophages is inhibited by IL-10 and the ligation of immune checkpoints PD-1 and signal regulatory protein α (SIRPα). MDSCs are assumed to release arginase I (Arg-I) and nitric oxide (NO). Cytotoxic activity of T cells is inhibited by TGF-β, Arg-I, and NO. TGF-β and Arg-I facilitate the trans-differentiation of helper T cells to T_regs_ in the tumor.

The QSP model includes pharmacokinetics and pharmacodynamics of the anti–PD-1 antibody pembrolizumab. The antibody is directly administered into the central compartment mimicking intravenous infusion, and the pharmacokinetics incorporates antibody clearance from the central compartment, transport between central and peripheral/tumor compartments, and the transport from tumor to tumor-draining lymph node compartments. Pharmacodynamics of anti–PD-1 antibody is modeled by the binding of the antibody to PD-1 receptor on cytotoxic T cells and macrophages, which blocks the interactions of PD-1 with PD-L1 and PD-L2. Subsequent reduction in the amount of ligand-bound PD-1 decreases the inhibitory action of PD-1 on T cell and macrophage-mediated killing/phagocytosis of cancer cells that are both modeled as Hill functions.

#### Extensions to clonal diversity and neo-epitopes

To account for the clonal heterogeneity of individual tumors, five cancer clones are considered. Apart from the number of cells of each cancer clone, individual cancer clones are assumed to differ in growth rate and neo-epitope expression. The presence of eight neo-epitopes (*70*) and cytotoxic T cells with different specificities corresponding to each neo-epitope is considered. Assuming that each neo-epitope is recognized by multiple TCR clonotypes, cytotoxic T cell population specific to each neo-epitope is assumed to consist of multiple T cell clonotypes. We also assume that cytotoxic T cells of any neo-epitope specificity can recognize and kill every cancer clone; however, the number of activated cytotoxic T cells specific to each epitope is dependent on the concentration of corresponding peptides presented by mature APCs.

### Model calibration

Model parameters were estimated in the previous versions of the QSP model using in vitro, preclinical, and clinical data or directly obtained from the literature. Tumor growth parameters were estimated by fitting the model to tumor growth curve from TNBC xenograft models and were scaled up to humans by allometric scaling. The initial amount of naïve T cells is based on the measurement from healthy individuals, and the rate of thymic export of naïve T cells is estimated using average ages of patients with breast cancer. Recruitment rate constants of immune cells are estimated to fit the cell density measurements from breast cancer samples. Cell surface expression of checkpoint molecules is obtained from flow cytometry measurements. Secretion of soluble factors such as cytokines is calibrated using the cytokine concentration in breast cancer samples. Expression levels of Arg-I and NO are estimated using in vitro data from breast cancer cells. Pharmacokinetic parameters of therapeutic antibody are estimated to fit the clinical measurements of plasma concentration. These parameters were inherited into the extended QSP model and left unchanged.

However, immune cell migration rate constants and cellular densities for the metastatic tumors are calibrated such that the immune cell content of metastatic tumors is consistent with cell abundance estimations from publicly available transcriptomic data as discussed in the “Calibration of the immune cell content of metastases” section. Parameters such as the seeding time of metastatic tumors, the initial number of cancer cells in tumor compartments, the number of antigen-specific T cell clones, the rate of T cell exhaustion by cancer cells, and the initial diameter of metastases were estimated to fit the clinical response data from KEYNOTE-119 trial as explained in the “Virtual clinical trial” section. The list of model parameters and references are provided in data S1.

### Estimation of the tumor microenvironment composition

RNA sequencing (RNA-seq) data generated by Huang *et al.* (*11*) and Siegel *et al.* (*71*) were obtained from the National Center for Biotechnology Information Gene Expression Omnibus (GEO) database (www.ncbi.nlm.nih.gov/geo/; GEO accession numbers GSE184717 and GSE110590, respectively). The abundance of immune cells in the tumor microenvironment was estimated from both datasets using MCP-counter (*72*), EPIC (*73*), and quanTIseq (*74*) using http://timer.cistrome.org/ (*75*–*77*).

The Huang dataset included transcriptomes of metastatic tumors from different sites of a TNBC patient and was used for the comparison of immune cell abundance between metastatic tumors in the lung, liver, brain, and bone (see figs. S1A and S2, A and B). After excluding low-purity samples [supplementary text of (*11*)], this dataset included seven lung, four liver, three brain, and three bone metastatic tumor samples. The Siegel dataset included transcriptomes of 39 metastatic tumors from sites such as the lung, liver, brain, bone, kidney, skin, adrenal and soft tissue, and matched primary tumors from nine patients with TNBC. We used a gene signature–based cell composition inference tool called xCell (*54*) to calculate immune and stroma score that provide the overall abundance of immune cells and stromal cells, respectively, in each sample using transcriptomic data.

### Calibration of the immune cell content of metastases

For the QSP model calibration, only the abundance estimates of EPIC and quanTIseq were used as these estimates can be interpreted as cell fractions/absolute cell counts (*76*) and can be directly compared with cell fractions from the simulations. The relative abundance of different cell types in metastatic tumors was calculated with respect to the matched primary tumor from the simulation results and compared with the relative abundance estimates from transcriptomic data. All metastatic tumor samples excluding the lung, brain, and lymph nodes were lumped together to estimate the immune cell composition of other metastatic tumors. Model parameters such as the recruitment rate constants of macrophages and MDSCs, the polarization rate constant of M1 to M2 macrophages, and the baseline density of APCs were estimated such that the abundance of cell types from simulations was consistent with estimates from the RNA-seq data. We use the relative abundance of immune cells in metastases with respect to primary tumor as the relative abundance estimates are expected to be less sensitive to the gene expression platform and normalization techniques used (*76*).

The transcriptomic data used to estimate immune cell abundances were collected from metastatic tumor samples from patients heavily pretreated with different chemotherapies. By calibrating the immune cell composition of metastases before the start of pembrolizumab treatment using these estimates, we assume that metastatic tumors in the model are heavily pretreated with chemotherapies as in the patients enrolled in KEYNOTE-119 trial (*22*). Thus, the effect of pretreatment with chemotherapies on immune cell composition is considered without the explicit simulation with chemotherapies in the model.

### Virtual clinical trial

Virtual patients were generated by Latin hypercube sampling of selected parameters, such as the seeding time of metastatic tumors, the initial tumor diameter, and the growth rate constant of cancer clones, from a chosen distribution (data S1); each parameter set corresponds to a virtual patient. Parameters randomly sampled account for intraindividual variability (tumor-to-tumor heterogeneity) in addition to the interindividual variability considered in previous studies (*33*, *36*). For each virtual patient, a few cancer cells were introduced into metastatic tumor compartments at randomly generated seeding time points, and the simulation was continued until a metastatic tumor reached the initial target diameter. Values of model species at the end of the simulation were used as initial conditions for treatment simulations. Among the 1000 virtual patients generated, only the virtual patients (816 of 1000) who met the target tumor diameter were considered for treatment simulations. Parameter sets corresponding to virtual patients who did not attain the target tumor diameter were discarded to eliminate potentially unrealistic virtual patients. After the start of virtual treatment, no metastatic tumors were seeded. This results in different number of tumors in simulated virtual patients despite the presence of three metastatic tumor compartments in all virtual patients. While virtual patients were filtered only on the basis of the ability to develop a tumor in this study, the availability of more clinical data would enable filtering virtual patients based on plausible ranges of species concentrations (*78*).

The virtual clinical trial simulation was performed by administering pembrolizumab at a dose of 200 mg every 3 weeks following the pembrolizumab arm of the KEYNOTE-119 trial (*22*). Assuming that most patients underwent primary tumor resection before enrollment in the KEYNOTE-119 trial, the initial number of cancer cells in the primary tumor compartment was set to 0 in all virtual patients.

Response of each virtual patient was categorized as complete response (CR)/PR, SD, or PD based on the RECIST v1.1 criteria (*67*). As in the KEYNOTE-119 clinical trial, a minimum duration of 24 weeks was considered for SD, and time intervals between subsequent observations for response evaluation were set to 9 weeks. Proportion of patients with CR/PR was considered as overall response rate. Only virtual patients who achieved a CR or PR were included in the calculation of the time to response and duration of response. Duration of response in patients who did not relapse until the end of simulation was set as the duration from response to the end of simulation. The total number of patients used in response rate calculations reported from the KEYNOTE-119 trial also includes nonevaluable patients (*22*). Thus, we renormalized the results from KEYNOTE-119 by excluding nonevaluable patients to enable direct comparison with the virtual clinical trial. Bootstrap sampling was performed to calculate 95% confidence intervals for response rates, duration of response, and time to response of the virtual patient population. We assumed that the percentage of patients with lung metastases in pembrolizumab arm is 65%, which is the percentage of patients with lung metastases in KEYNOTE-119 trial at baseline (*27*). Median seeding time of metastatic tumors was estimated to fit the percentage of patients with lung metastases in the virtual clinical trial. Because of the lack of data on the baseline metastatic tumor diameter, this parameter was estimated to fit the response rates resulting in a median tumor diameter of 1.65 cm that is within clinically reported ranges of the sizes of metastatic tumors (*11*).

### Biomarker analysis

We classified the virtual patients as responders and nonresponders based on the response status. In the analysis of treatment effects and biomarkers, patients demonstrating CR/PR or SD were considered as responders, whereas nonresponders included patients with PD. To identify and evaluate biomarkers, we considered two different metrics in this study: (i) response probability defined as the number of responders in a chosen patient subgroup divided by the total number of patients in the subgroup; (ii) RIS is defined as follows

$$\mathrm{RIS}=\frac{\text{No. of responders in a subgroup}}{\text{Total no. of responders in the entire cohort}}-\frac{\text{No. of nonresponders in a subgroup}}{\text{Total no. of nonresponders in the entire cohort}}$$ (1)

We selected densities/concentrations of different cellular and molecular species from central, lymph node, and tumor compartments as biomarker candidates. In addition, we also considered derived quantities such as the ratio of M1 and M2 macrophages. Taking advantage of the consideration of multiple cancer clones, neo-antigens, and cytotoxic T cell specificities in the QSP model, we considered diversity indices (*79*, *80*) of cancer cells and cytotoxic T cells as biomarker candidates.

Richness (*S*) is defined as the number of unique cancer clones or unique neo-antigen specificities among cytotoxic T cells. Shannon index (*H*) and evenness (*J*) are calculated using the following equations: *k* represents different clones for cancer cells or different neo-antigen specificities for cytotoxic T cells; *p_k_* represents proportion of cancer cells belonging to clone *k* or proportion of cytotoxic T cells with neo-antigen specificity *k*

$$H=-\sum_{k=1}^{S}p_{k}\ln(p_{k})$$ (2)

$$J=\frac{H}{\ln(S)}$$ (3)

For biomarker candidates from tumor or tumor-draining lymph node compartments, average values from all simulated metastatic tumors or tumor-draining lymph nodes, respectively, were calculated. For each biomarker candidate, the range of baseline level was determined in virtual patients previously generated for treatment simulations, and eight uniformly spaced threshold values/cutoffs were chosen, spanning the entire range of biomarker levels. Virtual patients with biomarker candidate values above and below chosen threshold values were selected as different subgroups. This results in virtual patient subgroups that are not mutually exclusive. Subgroups with total number of patients less than 20 were discarded. For each subgroup of virtual patients, response probability and RIS were calculated. Subgroup with highest response probability and RIS was considered as the best subgroup for each biomarker candidate. Biomarker candidates were ranked separately on the basis of the values of the two metrics, response probability and RIS, to identify potential biomarkers. To identify combinations of two biomarkers, all possible combinations of two biomarker candidates were generated. For each biomarker candidate combination, virtual patient subgroups were chosen on the basis of threshold values of both biomarker candidates. Biomarker combinations were then ranked on the basis of the highest response probability or RIS achieved in the best subgroup identified. Biomarker threshold values in virtual patient subgroups with highest response probability and RIS are provided in data S2.

## Notes

View/request a protocol for this paper from *Bio-protocol*.

## References

[1] D. Hanahan, Hallmarks of cancer: New dimensions. Cancer Discov. 12, 31–46 (2022). 35022204 10.1158/2159-8290.CD-21-1059

[2] P. S. Steeg, Tumor metastasis: Mechanistic insights and clinical challenges. Nat. Med. 12, 895–904 (2006). 16892035 10.1038/nm1469

[3] J. Fares, M. Y. Fares, H. H. Khachfe, H. A. Salhab, Y. Fares, Molecular principles of metastasis: A hallmark of cancer revisited. Signal Transduct. Target. Ther. 5, 28 (2020). 32296047 10.1038/s41392-020-0134-x PMC7067809

[4] A. C. Obenauf, J. Massagué, Surviving at a distance: Organ-specific metastasis. Trends Cancer 1, 76–91 (2015). 28741564 10.1016/j.trecan.2015.07.009 PMC4673677

[5] J. Budczies, M. von Winterfeld, F. Klauschen, M. Bockmayr, J. K. Lennerz, C. Denkert, T. Wolf, A. Warth, M. Dietel, I. Anagnostopoulos, W. Weichert, D. Wittschieber, A. Stenzinger, The landscape of metastatic progression patterns across major human cancers. Oncotarget 6, 570–583 (2015). 25402435 10.18632/oncotarget.2677 PMC4381616

[6] C. Anders, L. A. Carey, Understanding and treating triple-negative breast cancer. Oncology. (Williston Park) 22, 1233–1239 (2008). 18980022 PMC2868264

[7] N. U. Lin, E. Claus, J. Sohl, A. R. Razzak, A. Arnaout, E. P. Winer, Sites of distant recurrence and clinical outcomes in patients with metastatic triple-negative breast cancer: High incidence of central nervous system metastases. Cancer 113, 2638–2645 (2008). 18833576 10.1002/cncr.23930 PMC2835546

[8] K. Pogoda, A. Niwińska, M. Murawska, T. Pieńkowski, Analysis of pattern, time and risk factors influencing recurrence in triple-negative breast cancer patients. Med. Oncol. 30, 338 (2013). 23292831 10.1007/s12032-012-0388-4 PMC3586394

[9] L. A. Carey, E. C. Dees, L. Sawyer, L. Gatti, D. T. Moore, F. Collichio, D. W. Ollila, C. I. Sartor, M. L. Graham, C. M. Perou, The triple negative paradox: Primary tumor chemosensitivity of breast cancer subtypes. Clin. Cancer Res. 13, 2329–2334 (2007). 17438091 10.1158/1078-0432.CCR-06-1109

[10] W. D. Foulkes, I. E. Smith, J. S. Reis-Filho, Triple-negative breast cancer. N. Engl. J. Med. 363, 1938–1948 (2010). 21067385 10.1056/NEJMra1001389

[11] X. Huang, Y. Qiao, S. W. Brady, R. E. Factor, E. Downs-Kelly, A. Farrell, J. A. McQuerry, G. Shrestha, D. Jenkins, W. E. Johnson, A. L. Cohen, A. H. Bild, G. T. Marth, Novel temporal and spatial patterns of metastatic colonization from breast cancer rapid-autopsy tumor biopsies. Genome Med. 13, 170 (2021). 34711268 10.1186/s13073-021-00989-6 PMC8555066

[12] B. Szekely, V. Bossuyt, X. Li, V. B. Wali, G. A. Patwardhan, C. Frederick, A. Silber, T. Park, M. Harigopal, V. Pelekanou, M. Zhang, Q. Yan, D. L. Rimm, G. Bianchini, C. Hatzis, L. Pusztai, Immunological differences between primary and metastatic breast cancer. Ann. Oncol. 29, 2232–2239 (2018). 30203045 10.1093/annonc/mdy399

[13] A. Cimino-Mathews, X. Ye, A. Meeker, P. Argani, L. A. Emens, Metastatic triple-negative breast cancers at first relapse have fewer tumor-infiltrating lymphocytes than their matched primary breast tumors: A pilot study. Hum. Pathol. 44, 2055–2063 (2013). 23701942 10.1016/j.humpath.2013.03.010 PMC3758372

[14] L. Zhu, J. L. Narloch, S. Onkar, M. Joy, G. Broadwater, C. Luedke, A. Hall, R. Kim, K. Pogue-Geile, S. Sammons, N. Nayyar, U. Chukwueke, P. K. Brastianos, C. K. Anders, A. C. Soloff, D. A. A. Vignali, G. C. Tseng, L. A. Emens, P. C. Lucas, K. L. Blackwell, S. Oesterreich, A. V. Lee, Metastatic breast cancers have reduced immune cell recruitment but harbor increased macrophages relative to their matched primary tumors. J. Immunother. Cancer 7, 265 (2019). 31627744 10.1186/s40425-019-0755-1 PMC6798422

[15] A. Cimino-Mathews, E. Thompson, J. M. Taube, X. Ye, Y. Lu, A. Meeker, H. Xu, R. Sharma, K. Lecksell, T. C. Cornish, N. Cuka, P. Argani, L. A. Emens, PD-L1 (B7-H1) expression and the immune tumor microenvironment in primary and metastatic breast carcinomas. Hum. Pathol. 47, 52–63 (2016). 26527522 10.1016/j.humpath.2015.09.003 PMC4778421

[16] P. Aftimos, M. Oliveira, A. Irrthum, D. Fumagalli, C. Sotiriou, E. N. Gal-Yam, M. E. Robson, J. Ndozeng, A. Di Leo, E. M. Ciruelos, E. de Azambuja, G. Viale, E. D. Scheepers, G. Curigliano, J. M. Bliss, J. S. Reis-Filho, M. Colleoni, M. Balic, F. Cardoso, J. Albanell, C. Duhem, S. Marreaud, D. Romagnoli, B. Rojas, A. Gombos, H. Wildiers, A. Guerrero-Zotano, P. Hall, A. Bonetti, K. F. Larsson, M. Degiorgis, S. Khodaverdi, R. Greil, Á. Sverrisdóttir, M. Paoli, E. Seyll, S. Loibl, B. Linderholm, G. Zoppoli, N. E. Davidson, O. T. Johannsson, P. L. Bedard, S. Loi, S. Knox, D. A. Cameron, N. Harbeck, M. L. Montoya, M. Brandão, A. Vingiani, C. Caballero, F. S. Hilbers, L. R. Yates, M. Benelli, D. Venet, M. J. Piccart, Genomic and transcriptomic analyses of breast cancer primaries and matched metastases in Aurora, the breast international group (Big) molecular screening initiative. Cancer Discov. 11, 2796–2811 (2021). 34183353 10.1158/2159-8290.CD-20-1647 PMC9414283

[17] G. V. Echeverria, E. Powell, S. Seth, Z. Ge, A. Carugo, C. Bristow, M. Peoples, F. Robinson, H. Qiu, J. Shao, S. L. Jeter-Jones, X. Zhang, V. Ramamoorthy, S. Cai, W. Wu, G. Draetta, S. L. Moulder, W. F. Symmans, J. T. Chang, T. P. Heffernan, H. Piwnica-Worms, High-resolution clonal mapping of multi-organ metastasis in triple negative breast cancer. Nat. Commun. 9, 5079 (2018). 30498242 10.1038/s41467-018-07406-4 PMC6265294

[18] S. L. Topalian, G. J. Weiner, D. M. Pardoll, Cancer immunotherapy comes of age. J. Clin. Oncol. 29, 4828–4836 (2011). 22042955 10.1200/JCO.2011.38.0899 PMC3255990

[19] P. Darvin, S. M. Toor, V. S. Nair, E. Elkord, Immune checkpoint inhibitors: Recent progress and potential biomarkers. Exp. Mol. Med. 50, 1–11 (2018). 10.1038/s12276-018-0191-1 PMC6292890 30546008

[20] S. Adams, S. Loi, D. Toppmeyer, D. W. Cescon, M. De Laurentiis, R. Nanda, E. P. Winer, H. Mukai, K. Tamura, A. Armstrong, M. C. Liu, H. Iwata, L. Ryvo, P. Wimberger, H. S. Rugo, A. R. Tan, L. Jia, Y. Ding, V. Karantza, P. Schmid, Pembrolizumab monotherapy for previously untreated, PD-L1-positive, metastatic triple-negative breast cancer: Cohort B of the phase II KEYNOTE-086 study. Ann. Oncol. 30, 405–411 (2019). 30475947 10.1093/annonc/mdy518

[21] R. Nanda, L. Q. M. Chow, E. C. Dees, R. Berger, S. Gupta, R. Geva, L. Pusztai, K. Pathiraja, G. Aktan, J. D. Cheng, V. Karantza, L. Buisseret, Pembrolizumab in patients with advanced triple-negative breast cancer: Phase Ib keynote-012 study. J. Clin. Oncol. 34, 2460–2467 (2016). 27138582 10.1200/JCO.2015.64.8931 PMC6816000

[22] E. P. Winer, O. Lipatov, S.-A. Im, A. Goncalves, E. Muñoz-Couselo, K. S. Lee, P. Schmid, K. Tamura, L. Testa, I. Witzel, S. Ohtani, N. Turner, S. Zambelli, N. Harbeck, F. Andre, R. Dent, X. Zhou, V. Karantza, J. Mejia, J. Cortes; KEYNOTE-119 investigators, Pembrolizumab versus investigator-choice chemotherapy for metastatic triple-negative breast cancer (KEYNOTE-119): A randomised, open-label, phase 3 trial. Lancet Oncol. 22, 499–511 (2021). 33676601 10.1016/S1470-2045(20)30754-3

[23] S. Adams, P. Schmid, H. S. Rugo, E. P. Winer, D. Loirat, A. Awada, D. W. Cescon, H. Iwata, M. Campone, R. Nanda, R. Hui, G. Curigliano, D. Toppmeyer, J. O’Shaughnessy, S. Loi, S. Paluch-Shimon, A. R. Tan, D. Card, J. Zhao, V. Karantza, J. Cortés, Pembrolizumab monotherapy for previously treated metastatic triple-negative breast cancer: Cohort A of the phase II KEYNOTE-086 study. Ann. Oncol. 30, 397–404 (2019). 30475950 10.1093/annonc/mdy517

[24] P. Schmid, J. Cortes, R. Dent, L. Pusztai, H. McArthur, S. Kümmel, J. Bergh, C. Denkert, Y. H. Park, R. Hui, N. Harbeck, M. Takahashi, M. Untch, P. A. Fasching, F. Cardoso, J. Andersen, D. Patt, M. Danso, M. Ferreira, M.-A. Mouret-Reynier, S.-A. Im, J.-H. Ahn, M. Gion, S. Baron-Hay, J.-F. Boileau, Y. Ding, K. Tryfonidis, G. Aktan, V. Karantza, J. O’Shaughnessy; KEYNOTE-522 Investigators, Event-free survival with pembrolizumab in early triple-negative breast cancer. N. Engl. J. Med. 386, 556–567 (2022). 35139274 10.1056/NEJMoa2112651

[25] J. Cortes, D. W. Cescon, H. S. Rugo, Z. Nowecki, S.-A. Im, M. M. Yusof, C. Gallardo, O. Lipatov, C. H. Barrios, E. Holgado, H. Iwata, N. Masuda, M. T. Otero, E. Gokmen, S. Loi, Z. Guo, J. Zhao, G. Aktan, V. Karantza, P. Schmid; KEYNOTE-355 Investigators, Pembrolizumab plus chemotherapy versus placebo plus chemotherapy for previously untreated locally recurrent inoperable or metastatic triple-negative breast cancer (KEYNOTE-355): A randomised, placebo-controlled, double-blind, phase 3 clinical trial. Lancet 396, 1817–1828 (2020). 33278935 10.1016/S0140-6736(20)32531-9

[26] J. Cortes, H. S. Rugo, D. W. Cescon, S.-A. Im, M. M. Yusof, C. Gallardo, O. Lipatov, C. H. Barrios, J. Perez-Garcia, H. Iwata, N. Masuda, M. T. Otero, E. Gokmen, S. Loi, Z. Guo, X. Zhou, V. Karantza, W. Pan, P. Schmid; KEYNOTE-355 Investigators, Pembrolizumab plus chemotherapy in advanced triple-negative breast cancer. N. Engl. J. Med. 387, 217–226 (2022). 35857659 10.1056/NEJMoa2202809

[27] E. Winer, O. Lipatov, S.-A. Im, A. Goncalves, K. S. Lee, P. Schmid, L. Testa, I. Witzel, S. Ohtani, N. Turner, S. Zambelli, N. Harbeck, F. Andre, R. Dent, J. Lin, V. Karantza, J. Mejia, J. Cortes, Abstract PS12-01: Pembrolizumab versus chemotherapy for previously treated metastatic triple-negative breast cancer (KEYNOTE-119): Efficacy in patients with lung or liver metastases. Cancer Res. 81, PS12-01 (2021).

[28] V. R. Knight-Schrijver, V. Chelliah, L. Cucurull-Sanchez, N. Le Novère, The promises of quantitative systems pharmacology modelling for drug development. Comput. Struct. Biotechnol. J. 14, 363–370 (2016). 27761201 10.1016/j.csbj.2016.09.002 PMC5064996

[29] K. Azer, C. D. Kaddi, J. S. Barrett, J. P. F. Bai, S. T. McQuade, N. J. Merrill, B. Piccoli, S. Neves-Zaph, L. Marchetti, R. Lombardo, S. Parolo, S. R. C. Immanuel, N. S. Baliga, History and future perspectives on the discipline of quantitative systems pharmacology modeling and its applications. Front. Physiol. 12, 637999 (2021). 33841175 10.3389/fphys.2021.637999 PMC8027332

[30] V. Chelliah, G. Lazarou, S. Bhatnagar, J. P. Gibbs, M. Nijsen, A. Ray, B. Stoll, R. A. Thompson, A. Gulati, S. Soukharev, A. Yamada, J. Weddell, H. Sayama, M. Oishi, S. Wittemer-Rump, C. Patel, C. Niederalt, R. Burghaus, C. Scheerans, J. Lippert, S. Kabilan, I. Kareva, N. Belousova, A. Rolfe, A. Zutshi, M. Chenel, F. Venezia, S. Fouliard, H. Oberwittler, A. Scholer-Dahirel, H. Lelievre, D. Bottino, S. C. Collins, H. Q. Nguyen, H. Wang, T. Yoneyama, A. Z. X. Zhu, P. H. van der Graaf, A. M. Kierzek, Quantitative systems pharmacology approaches for immuno-oncology: Adding virtual patients to the development paradigm. Clin. Pharmacol. Ther. 109, 605–618 (2021). 32686076 10.1002/cpt.1987 PMC7983940

[31] H. Ma, H. Wang, R. J. Sove, M. Jafarnejad, C.-H. Tsai, J. Wang, C. Giragossian, A. S. Popel, A quantitative systems pharmacology model of T cell engager applied to solid tumor. AAPS J. 22, 85 (2020). 32533270 10.1208/s12248-020-00450-3 PMC7293198

[32] H. Ma, H. Wang, R. J. Sové, J. Wang, C. Giragossian, A. S. Popel, Combination therapy with T cell engager and PD-L1 blockade enhances the antitumor potency of T cells as predicted by a QSP model. J. Immunother. Cancer 8, e001141 (2020). 32859743 10.1136/jitc-2020-001141 PMC7454244

[33] H. Wang, C. Zhao, C. A. Santa-Maria, L. A. Emens, A. S. Popel, Dynamics of tumor-associated macrophages in a quantitative systems pharmacology model of immunotherapy in triple-negative breast cancer. iScience 25, 104702 (2022). 35856032 10.1016/j.isci.2022.104702 PMC9287616

[34] H. Wang, R. J. Sové, M. Jafarnejad, S. Rahmeh, E. M. Jaffee, V. Stearns, E. T. R. Torres, R. M. Connolly, A. S. Popel, Conducting a virtual clinical trial in HER2-negative breast cancer using a quantitative systems pharmacology model with an epigenetic modulator and immune checkpoint inhibitors. Front. Bioeng. Biotechnol. 8, 141 (2020). 32158754 10.3389/fbioe.2020.00141 PMC7051945

[35] H. Wang, O. Milberg, I. H. Bartelink, P. Vicini, B. Wang, R. Narwal, L. Roskos, C. A. Santa-Maria, A. S. Popel, In silico simulation of a clinical trial with anti-CTLA-4 and anti-PD-L1 immunotherapies in metastatic breast cancer using a systems pharmacology model. R. Soc. Open Sci. 6, 190366 (2019). 31218069 10.1098/rsos.190366 PMC6549962

[36] H. Wang, H. Ma, R. J. Sové, L. A. Emens, A. S. Popel, Quantitative systems pharmacology model predictions for efficacy of atezolizumab and nab-paclitaxel in triple-negative breast cancer. J. Immunother. Cancer 9, e002100 (2021). 33579739 10.1136/jitc-2020-002100 PMC7883871

[37] R. Coletti, L. Leonardelli, S. Parolo, L. Marchetti, A QSP model of prostate cancer immunotherapy to identify effective combination therapies. Sci. Rep. 10, 9063 (2020). 32493951 10.1038/s41598-020-65590-0 PMC7270132

[38] M. Jafarnejad, C. Gong, E. Gabrielson, I. H. Bartelink, P. Vicini, B. Wang, R. Narwal, L. Roskos, A. S. Popel, A computational model of neoadjuvant PD-1 inhibition in non-small cell lung cancer. AAPS J. 21, 79 (2019). 31236847 10.1208/s12248-019-0350-x PMC6591205

[39] O. Milberg, C. Gong, M. Jafarnejad, I. H. Bartelink, B. Wang, P. Vicini, R. Narwal, L. Roskos, A. S. Popel, A QSP model for predicting clinical responses to monotherapy, combination and sequential therapy following CTLA-4, PD-1, and PD-L1 checkpoint blockade. Sci. Rep. 9, 11286 (2019). 31375756 10.1038/s41598-019-47802-4 PMC6677731

[40] R. Kumar, K. Thiagarajan, L. Jagannathan, L. Liu, K. Mayawala, D. de Alwis, B. Topp, Beyond the single average tumor: Understanding IO combinations using a clinical QSP model that incorporates heterogeneity in patient response. CPT Pharmacometrics Syst. Pharmacol. 10, 684–695 (2021). 33938166 10.1002/psp4.12637 PMC8302246

[41] J. Zhang, Z. Ji, J. X. Caushi, M. El Asmar, V. Anagnostou, T. R. Cottrell, H. Y. Chan, P. Suri, H. Guo, T. Merghoub, J. E. Chaft, J. E. Reuss, A. J. Tam, R. L. Blosser, M. Abu-Akeel, J.-W. Sidhom, N. Zhao, J. S. Ha, D. R. Jones, K. A. Marrone, J. Naidoo, E. Gabrielson, J. M. Taube, V. E. Velculescu, J. R. Brahmer, F. Housseau, M. D. Hellmann, P. M. Forde, D. M. Pardoll, H. Ji, K. N. Smith, Compartmental analysis of T-cell clonal dynamics as a function of pathologic response to neoadjuvant PD-1 blockade in resectable non–small cell lung cancer. Clin. Cancer Res. 26, 1327–1337 (2020). 31754049 10.1158/1078-0432.CCR-19-2931 PMC7073288

[42] S. Loi, E. Winer, O. Lipatov, S.-A. Im, A. Goncalves, J. Cortes, K. S. Lee, P. Schmid, L. Testa, I. Witzel, S. Ohtani, N. Turner, S. Zambelli, N. Harbeck, F. Andre, R. Dent, L. Huang, J. Mejia, V. Karantza, R. Salgado, Abstract PD5-03: Relationship between tumor-infiltrating lymphocytes (TILs) and outcomes in the KEYNOTE-119 study of pembrolizumab vs chemotherapy for previously treated metastatic triple-negative breast cancer (mTNBC). Cancer Res. 80, PD5-03 (2020).

[43] G. Lazarou, V. Chelliah, B. G. Small, M. Walker, P. H. van der Graaf, A. M. Kierzek, Integration of omics data sources to inform mechanistic modeling of immune-oncology therapies: A tutorial for clinical pharmacologists. Clin. Pharmacol. Ther. 107, 858–870 (2020). 31955413 10.1002/cpt.1786 PMC7158209

[44] Q. Tan, S. Yin, D. Zhou, Y. Chi, X. Man, H. Li, Potential predictive and prognostic value of biomarkers related to immune checkpoint inhibitor therapy of triple-negative breast cancer. Front. Oncol. 12, 779786 (2022). 35646659 10.3389/fonc.2022.779786 PMC9134495

[45] J. Cortés, O. Lipatov, S.-A. Im, A. Gonçalves, K. S. Lee, P. Schmid, K. Tamura, L. Testa, I. Witzel, S. Ohtani, S. Zambelli, N. Harbeck, F. André, R. Dent, X. Zhou, V. Karantza, J. A. Mejia, E. P. Winer, KEYNOTE-119: Phase III study of pembrolizumab (pembro) versus single-agent chemotherapy (chemo) for metastatic triple negative breast cancer (mTNBC). Ann. Oncol. 30, v859–v860 (2019).

[46] L. Molinero, Y. Li, C.-W. Chang, S. Maund, M. Berg, J. Harrison, M. Fassò, C. O’Hear, P. Hegde, L. A. Emens, Tumor immune microenvironment and genomic evolution in a patient with metastatic triple negative breast cancer and a complete response to atezolizumab. J. Immunother. Cancer 7, 274 (2019). 31647026 10.1186/s40425-019-0740-8 PMC6813065

[47] L. A. Emens, C. Cruz, J. P. Eder, F. Braiteh, C. Chung, S. M. Tolaney, I. Kuter, R. Nanda, P. A. Cassier, J.-P. Delord, M. S. Gordon, E. ElGabry, C.-W. Chang, I. Sarkar, W. Grossman, C. O’Hear, M. Fassò, L. Molinero, P. Schmid, Long-term clinical outcomes and biomarker analyses of atezolizumab therapy for patients with metastatic triple-negative breast cancer: A phase 1 study. JAMA Oncol. 5, 74–82 (2019). 30242306 10.1001/jamaoncol.2018.4224 PMC6439773

[48] P. Schmid, S. Adams, H. S. Rugo, A. Schneeweiss, C. H. Barrios, H. Iwata, V. Diéras, R. Hegg, S.-A. Im, G. Shaw Wright, V. Henschel, L. Molinero, S. Y. Chui, R. Funke, A. Husain, E. P. Winer, S. Loi, L. A. Emens, Atezolizumab and nab-paclitaxel in advanced triple-negative breast cancer. N. Engl. J. Med. 379, 2108–2121 (2018). 30345906 10.1056/NEJMoa1809615

[49] S. García-Mulero, M. H. Alonso, J. Pardo, C. Santos, X. Sanjuan, R. Salazar, V. Moreno, J. M. Piulats, R. Sanz-Pamplona, Lung metastases share common immune features regardless of primary tumor origin. J. Immunother. Cancer 8, e000491 (2020). 32591432 10.1136/jitc-2019-000491 PMC7319789

[50] M. Wang, S. Wang, J. Desai, J. A. Trapani, P. J. Neeson, Therapeutic strategies to remodel immunologically cold tumors. Clin. Transl. Immunology 9, e1226 (2020). 35136604 10.1002/cti2.1226 PMC8809427

[51] P. Bonaventura, T. Shekarian, V. Alcazer, J. Valladeau-Guilemond, S. Valsesia-Wittmann, S. Amigorena, C. Caux, S. Depil, Cold tumors: A therapeutic challenge for immunotherapy. Front. Immunol. 10, 168 (2019). 30800125 10.3389/fimmu.2019.00168 PMC6376112

[52] J. C. Lee, S. Mehdizadeh, J. Smith, A. Young, I. A. Mufazalov, C. T. Mowery, A. Daud, J. A. Bluestone, Regulatory T cell control of systemic immunity and immunotherapy response in liver metastasis. Sci. Immunol. 5, eaba0759 (2020). 33008914 10.1126/sciimmunol.aba0759 PMC7755924

[53] J. Yu, M. D. Green, S. Li, Y. Sun, S. N. Journey, J. E. Choi, S. M. Rizvi, A. Qin, J. J. Waninger, X. Lang, Z. Chopra, I. El Naqa, J. Zhou, Y. Bian, L. Jiang, A. Tezel, J. Skvarce, R. K. Achar, M. Sitto, B. S. Rosen, F. Su, S. P. Narayanan, X. Cao, S. Wei, W. Szeliga, L. Vatan, C. Mayo, M. A. Morgan, C. A. Schonewolf, K. Cuneo, I. Kryczek, V. T. Ma, C. D. Lao, T. S. Lawrence, N. Ramnath, F. Wen, A. M. Chinnaiyan, M. Cieslik, A. Alva, W. Zou, Liver metastasis restrains immunotherapy efficacy via macrophage-mediated T cell elimination. Nat. Med. 27, 152–164 (2021). 33398162 10.1038/s41591-020-1131-x PMC8095049

[54] D. Aran, Z. Hu, A. J. Butte, xCell: Digitally portraying the tissue cellular heterogeneity landscape. Genome Biol. 18, 220 (2017). 29141660 10.1186/s13059-017-1349-1 PMC5688663

[55] M. Bartoschek, N. Oskolkov, M. Bocci, J. Lövrot, C. Larsson, M. Sommarin, C. D. Madsen, D. Lindgren, G. Pekar, G. Karlsson, M. Ringnér, J. Bergh, Å. Björklund, K. Pietras, Spatially and functionally distinct subclasses of breast cancer-associated fibroblasts revealed by single cell RNA sequencing. Nat. Commun. 9, 5150 (2018). 30514914 10.1038/s41467-018-07582-3 PMC6279758

[56] R. Kalluri, The biology and function of fibroblasts in cancer. Nat. Rev. Cancer 16, 582–598 (2016). 27550820 10.1038/nrc.2016.73

[57] M. K. Malone, K. Smrekar, S. Park, B. Blakely, A. Walter, N. Nasta, J. Park, M. Considine, L. V. Danilova, N. B. Pandey, E. J. Fertig, A. S. Popel, K. Jin, Cytokines secreted by stromal cells in TNBC microenvironment as potential targets for cancer therapy. Cancer Biol. Ther. 21, 560–569 (2020). 32213106 10.1080/15384047.2020.1739484 PMC7515526

[58] F. Posch, K. Silina, S. Leibl, A. Mündlein, H. Moch, A. Siebenhüner, P. Samaras, J. Riedl, M. Stotz, J. Szkandera, H. Stöger, M. Pichler, R. Stupp, M. van den Broek, P. Schraml, A. Gerger, U. Petrausch, T. Winder, Maturation of tertiary lymphoid structures and recurrence of stage II and III colorectal cancer. Oncoimmunology 7, e1378844 (2018). 29416939 10.1080/2162402X.2017.1378844 PMC5798199

[59] A. J. Gunderson, V. Rajamanickam, C. Bui, B. Bernard, J. Pucilowska, C. Ballesteros-Merino, M. Schmidt, K. McCarty, M. Philips, B. Piening, C. Dubay, T. Medler, P. Newell, P. Hansen, E. Tran, E. Tang, C. Bifulco, M. Crittenden, M. Gough, K. H. Young, Germinal center reactions in tertiary lymphoid structures associate with neoantigen burden, humoral immunity and long-term survivorship in pancreatic cancer. Oncoimmunology 10, 1900635 (2021). 33796412 10.1080/2162402X.2021.1900635 PMC7993148

[60] K. Siliņa, A. Soltermann, F. M. Attar, R. Casanova, Z. M. Uckeley, H. Thut, M. Wandres, S. Isajevs, P. Cheng, A. Curioni-Fontecedro, P. Foukas, M. P. Levesque, H. Moch, A. Linē, M. van den Broek, Germinal centers determine the prognostic relevance of tertiary lymphoid structures and are impaired by corticosteroids in lung squamous cell carcinoma. Cancer Res. 78, 1308–1320 (2018). 29279354 10.1158/0008-5472.CAN-17-1987

[61] S. Kordasti, B. Costantini, T. Seidl, P. Perez Abellan, M. Martinez Llordella, D. McLornan, K. E. Diggins, A. Kulasekararaj, C. Benfatto, X. Feng, A. Smith, S. A. Mian, R. Melchiotti, E. de Rinaldis, R. Ellis, N. Petrov, G. A. M. Povoleri, S. S. Chung, N. S. B. Thomas, F. Farzaneh, J. M. Irish, S. Heck, N. S. Young, J. C. W. Marsh, G. J. Mufti, Deep phenotyping of Tregs identifies an immune signature for idiopathic aplastic anemia and predicts response to treatment. Blood 128, 1193–1205 (2016). 27281795 10.1182/blood-2016-03-703702 PMC5009512

[62] M. Ouzounova, E. Lee, R. Piranlioglu, A. El Andaloussi, R. Kolhe, M. F. Demirci, D. Marasco, I. Asm, A. Chadli, K. A. Hassan, M. Thangaraju, G. Zhou, A. S. Arbab, J. K. Cowell, H. Korkaya, Monocytic and granulocytic myeloid derived suppressor cells differentially regulate spatiotemporal tumour plasticity during metastatic cascade. Nat. Commun. 8, 14979 (2017). 28382931 10.1038/ncomms14979 PMC5384228

[63] H. Mi, S. Sivagnanam, C. B. Betts, S. M. Liudahl, E. M. Jaffee, L. M. Coussens, A. S. Popel, Quantitative spatial profiling of immune populations in pancreatic ductal adenocarcinoma reveals tumor microenvironment heterogeneity and prognostic biomarkers. Cancer Res. 82, 4359–4372 (2022). 36112643 10.1158/0008-5472.CAN-22-1190 PMC9716253

[64] C. Gong, A. Ruiz-Martinez, H. Kimko, A. S. Popel, A spatial quantitative systems pharmacology platform spQSP-IO for simulations of tumor–immune interactions and effects of checkpoint inhibitor immunotherapy. Cancers (Basel) 13, 3751 (2021). 34359653 10.3390/cancers13153751 PMC8345161

[65] A. Ruiz-Martinez, C. Gong, H. Wang, R. J. Sové, H. Mi, H. Kimko, A. S. Popel, Simulations of tumor growth and response to immunotherapy by coupling a spatial agent-based model with a whole-patient quantitative systems pharmacology model. PLOS Comput. Biol. 18, e1010254 (2022). 35867773 10.1371/journal.pcbi.1010254 PMC9348712

[66] S. Zhang, C. Gong, A. Ruiz-Martinez, H. Wang, E. Davis-Marcisak, A. Deshpande, A. S. Popel, E. J. Fertig, Integrating single cell sequencing with a spatial quantitative systems pharmacology model spQSP for personalized prediction of triple-negative breast cancer immunotherapy response. Immunoinformatics (Amst) 1-2, 100002 (2021). 34708216 10.1016/j.immuno.2021.100002 PMC8547770

[67] E. A. Eisenhauer, P. Therasse, J. Bogaerts, L. H. Schwartz, D. Sargent, R. Ford, J. Dancey, S. Arbuck, S. Gwyther, M. Mooney, L. Rubinstein, L. Shankar, L. Dodd, R. Kaplan, D. Lacombe, J. Verweij, New response evaluation criteria in solid tumours: Revised RECIST guideline (version 1.1). Eur. J. Cancer 45, 228–247 (2009). 19097774 10.1016/j.ejca.2008.10.026

[68] H. Zhu, R. J. Melder, L. T. Baxter, R. K. Jain, Physiologically based kinetic model of effector cell biodistribution in mammals: Implications for adoptive immunotherapy. Cancer Res. 56, 3771–3781 (1996). 8706023

[69] J. M. Marchingo, A. Kan, R. M. Sutherland, K. R. Duffy, C. J. Wellard, G. T. Belz, A. M. Lew, M. R. Dowling, S. Heinzel, P. D. Hodgkin, Antigen affinity, costimulation, and cytokine inputs sum linearly to amplify T cell expansion. Science 346, 1123–1127 (2014). 25430770 10.1126/science.1260044

[70] P. Narang, M. Chen, A. A. Sharma, K. S. Anderson, M. A. Wilson, The neoepitope landscape of breast cancer: Implications for immunotherapy. BMC Cancer 19, 200 (2019). 30832597 10.1186/s12885-019-5402-1 PMC6399957

[71] M. B. Siegel, X. He, K. A. Hoadley, A. Hoyle, J. B. Pearce, A. L. Garrett, S. Kumar, V. J. Moylan, C. M. Brady, A. E. D. Van Swearingen, D. Marron, G. P. Gupta, L. B. Thorne, N. Kieran, C. Livasy, E. R. Mardis, J. S. Parker, M. Chen, C. K. Anders, L. A. Carey, C. M. Perou, Integrated RNA and DNA sequencing reveals early drivers of metastatic breast cancer. J. Clin. Invest. 128, 1371–1383 (2018). 29480819 10.1172/JCI96153 PMC5873890

[72] E. Becht, N. A. Giraldo, L. Lacroix, B. Buttard, N. Elarouci, F. Petitprez, J. Selves, P. Laurent-Puig, C. Sautès-Fridman, W. H. Fridman, A. de Reyniès, Estimating the population abundance of tissue-infiltrating immune and stromal cell populations using gene expression. Genome Biol. 17, 218 (2016). 27765066 10.1186/s13059-016-1070-5 PMC5073889

[73] J. Racle, K. de Jonge, P. Baumgaertner, D. E. Speiser, D. Gfeller, Simultaneous enumeration of cancer and immune cell types from bulk tumor gene expression data. eLife 6, e26476 (2017). 29130882 10.7554/eLife.26476 PMC5718706

[74] F. Finotello, C. Mayer, C. Plattner, G. Laschober, D. Rieder, H. Hackl, A. Krogsdam, Z. Loncova, W. Posch, D. Wilflingseder, S. Sopper, M. Ijsselsteijn, T. P. Brouwer, D. Johnson, Y. Xu, Y. Wang, M. E. Sanders, M. V. Estrada, P. Ericsson-Gonzalez, P. Charoentong, J. Balko, N. F. da Cunha Carvalho de Miranda, Z. Trajanoski, Molecular and pharmacological modulators of the tumor immune contexture revealed by deconvolution of RNA-seq data. Genome Med. 11, 34 (2019). 31126321 10.1186/s13073-019-0638-6 PMC6534875

[75] B. Li, E. Severson, J.-C. Pignon, H. Zhao, T. Li, J. Novak, P. Jiang, H. Shen, J. C. Aster, S. Rodig, S. Signoretti, J. S. Liu, X. S. Liu, Comprehensive analyses of tumor immunity: Implications for cancer immunotherapy. Genome Biol. 17, 174 (2016). 27549193 10.1186/s13059-016-1028-7 PMC4993001

[76] G. Sturm, F. Finotello, F. Petitprez, J. D. Zhang, J. Baumbach, W. H. Fridman, M. List, T. Aneichyk, Comprehensive evaluation of transcriptome-based cell-type quantification methods for immuno-oncology. Bioinformatics 35, i436–i445 (2019). 31510660 10.1093/bioinformatics/btz363 PMC6612828

[77] T. Li, J. Fu, Z. Zeng, D. Cohen, J. Li, Q. Chen, B. Li, X. S. Liu, TIMER2.0 for analysis of tumor-infiltrating immune cells. Nucleic Acids Res. 48, W509–W514 (2020). 32442275 10.1093/nar/gkaa407 PMC7319575

[78] R. J. Allen, T. R. Rieger, C. J. Musante, Efficient generation and selection of virtual populations in quantitative systems pharmacology models. CPT Pharmacometrics Syst. Pharmacol. 5, 140–146 (2016). 27069777 10.1002/psp4.12063 PMC4809626

[79] C. E. Shannon, A mathematical theory of communication. Bell Syst. Tech. J. 27, 379–423 (1948).

[80] E. C. Pielou, The measurement of diversity in different types of biological collections. J. Theor. Biol. 13, 131–144 (1966).

