# Supplementary material for: A transcriptome-informed QSP model of metastatic triple-negative breast cancer identifies predictive biomarkers for PD-1 inhibition
Source: Sci Adv. 2023 Jun 30;9(26):eadg0289. doi: 10.1126/sciadv.adg0289 (PMC10313177; doi:10.1126/sciadv.adg0289)
Supplement: Supplementary file 1 — Figs. S1 to S14 Legends for data S1 and S2 [file sciadv.adg0289_sm.pdf]

Science Advances
AAAS

Supplementary Materials for

A transcriptome-informed QSP model of metastatic triple-negative breast cancer identifies predictive biomarkers for PD-1 inhibition

Theinmozhi Arulraj *et al.*

Corresponding author: Theinmozhi Arulraj, tarulra1@jhmi.edu

Sci. Adv. **9**, eadg0289 (2023)
DOI: 10.1126/sciadv.adg0289

The PDF file includes:

Figs. S1 to S14
Legends for data S1 and S2

Other Supplementary Material for this manuscript includes the following:

Data S1 and S2

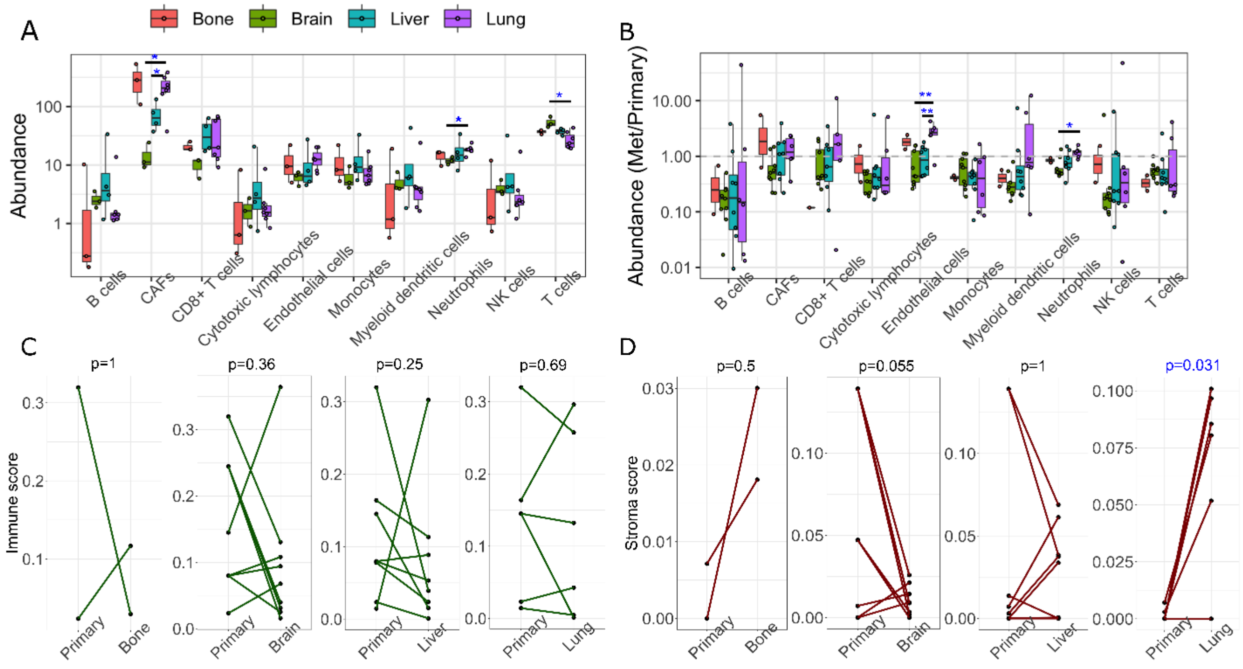

Figure S1: Cell type abundances of primary and metastatic TNBC tumors. (A) MCP-counter abundance estimates for different cell types in the microenvironment of metastatic tumors from a single TNBC patient. Metastatic tumors from different organs are shown in different colors. RNA-seq data from Huang et al. (*11*), was used in the analysis. **(B)** MCP-counter abundance estimates for different cell types expressed as ratio of primary and matched metastatic tumors. RNA-seq data from Siegel et al. (*71*). **(C)** and **(D)** xCell estimates of immune score and stroma score for matched primary and metastatic tumors. Statistical significance was computed by Wilcoxon test (* = p < 0.05, ** = p < 0.01).

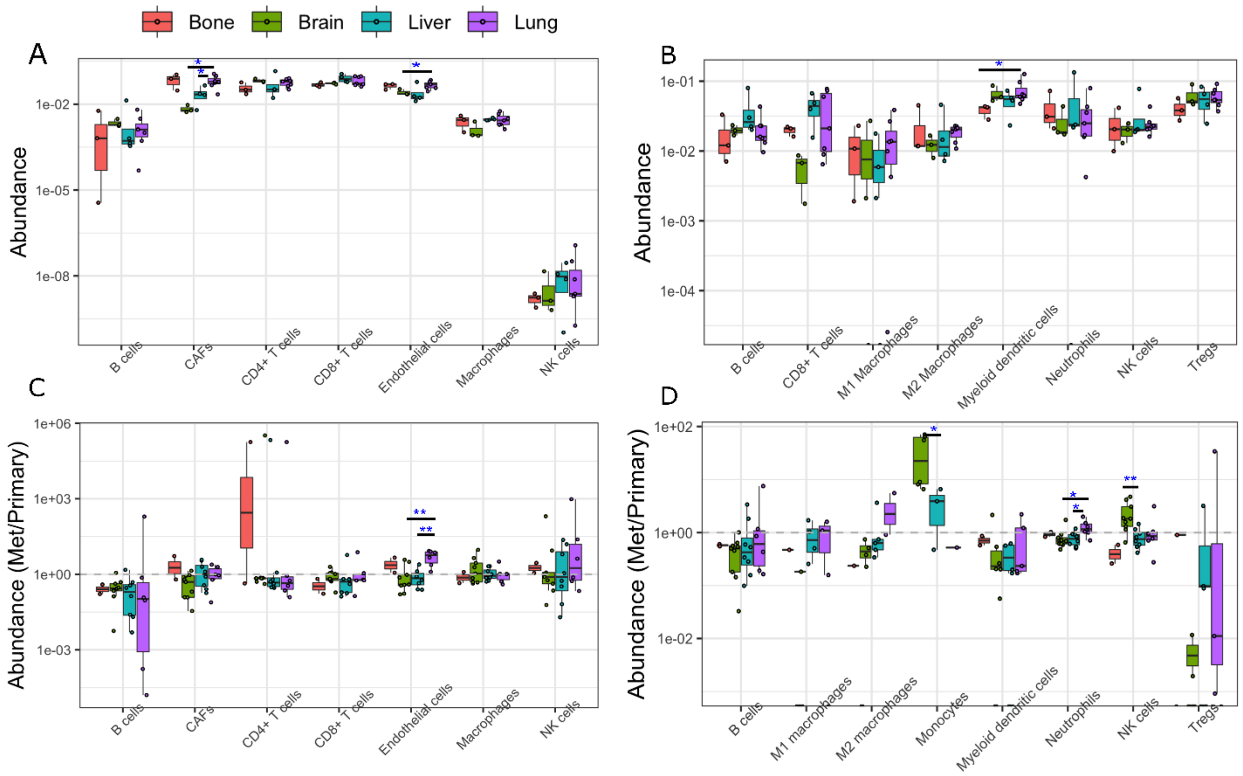

Figure S2: (A) and **(B)** Cell type abundances of metastatic TNBC tumors from different organs estimated using EPIC **(A)** and quanTIseq **(B)**. **(C)** and **(D)** Abundances of different cell types in metastatic tumors relative to matched primary tumors estimated using EPIC **(C)** and quanTIseq **(D)**. Data from Huang et al. (*11*) and Siegel et al (*71*). Metastatic tumors from different organs are shown in different colors. Statistical significance was computed by Wilcoxon test (* = p < 0.05, ** = p < 0.01)

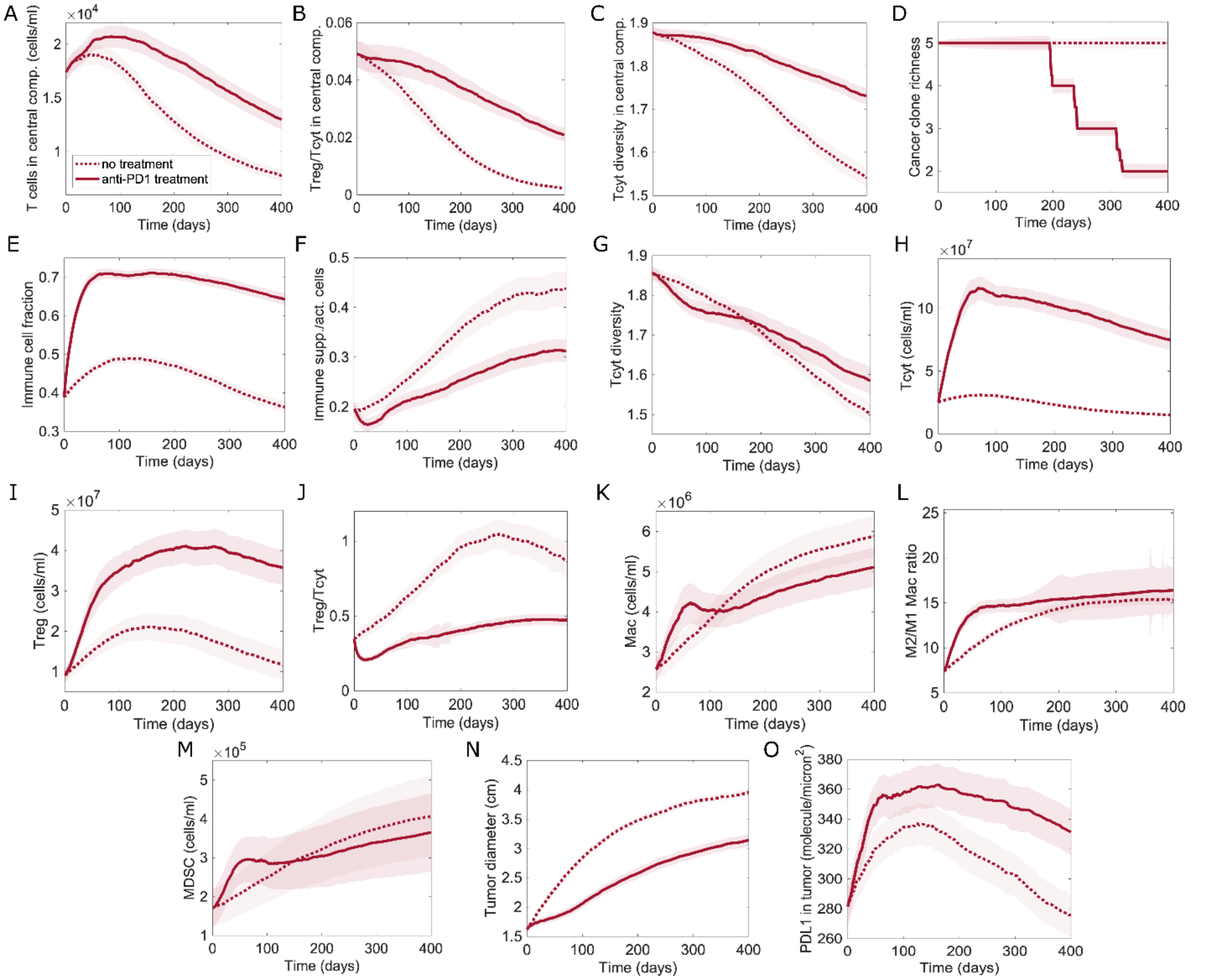

Figure S3: Time series of model species in virtual patients with or without treatment. (A) Density of activated T cells in the central compartment, **(B)** Ratio of regulatory and cytotoxic T cells (Tregs and Tcyt, respectively) in the central compartment, **(C)** Diversity of cytotoxic T cells in the central compartment, **(D)** Richness of cancer clones in the tumor, **(E)** Fraction of immune cells in the tumor, **(F)** Ratio of immune suppressive and immune activating cell densities in the tumor, **(G)** Diversity of cytotoxic T cells in the tumor, **(H)** Density of cytotoxic T cells in the tumor, **(I)** Density of regulatory T cells in the tumor, **(J)** Ratio of regulatory and cytotoxic T cell densities in the tumor, **(K)** Density of macrophages (Mac) in the tumor, **(L)** Ratio of M2 and M1 macrophage densities in the tumor, **(M)** Density of myeloid-derived suppressor cells in the tumor (MDSC), **(N)** Tumor diameter and **(O)** Expression of PD-L1 in the tumor. Quantities from tumor and lymph node compartments represent average values from all metastatic tumors simulated and lymph node compartments, respectively. Solid curves show median values of virtual patients. Shaded regions represent 95% confidence intervals.

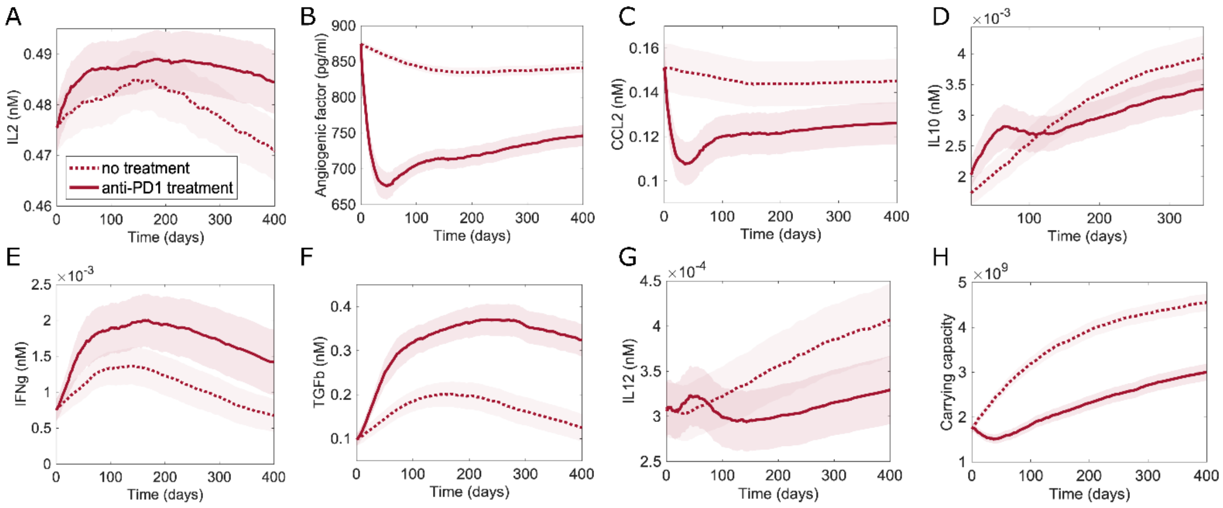

Figure S4: Time series of cytokines or other model species in virtual patients with or without treatment as in Figure S3. Concentrations of **(A)** IL-2 in the lymph node, **(B)** Angiogenic factor in the tumor, **(C)** CCL2 in the tumor, **(D)** IL-10 in the tumor, **(E)** IFN-γ in the tumor, **(F)** TGF-β in the tumor, **(G)** IL-12 in the tumor and **(H)** Carrying capacity of the tumor. Quantities from tumor and lymph node compartments represent average values from all metastatic tumors simulated and lymph node compartments, respectively. Solid curves show median values of virtual patients. Shaded regions represent 95% confidence intervals.

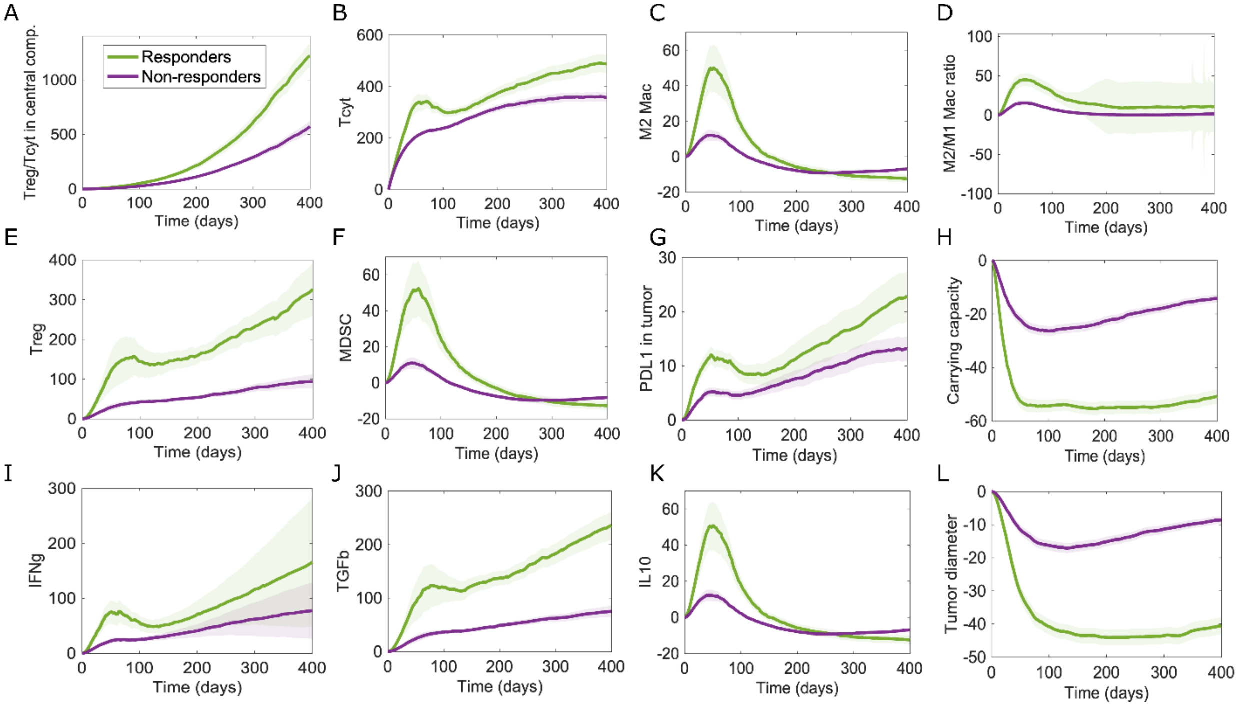

Figure S5: Effect of pembrolizumab monotherapy on model species in responders and non-responders as in Figure 4. Time series of percentage change in **(A)** Ratio of regulatory and cytotoxic T cells (Tregs and Tcyt, respectively) in the central compartment, **(B)** Density of cytotoxic T cells in the tumor, **(C)** Density of M2 macrophages (Mac) in the tumor, **(D)** Ratio of M2 and M1 macrophages density in the tumor, **(E)** Density of regulatory T cells in the tumor, **(F)** Density of myeloid-derived suppressor cells (MDSC) in the tumor, **(G)** Expression of PD-L1 in the tumor, **(H)** Tumor carrying capacity, **(I)** Concentration of IFN-γ in the tumor, **(J)** Concentration of TGF-β in the tumor, **(K)** Concentration of IL-10 in the tumor and **(L)** Tumor diameter, in treatment simulations of virtual patients with respect to simulations of same virtual patient cohort without any treatment. Quantities from tumor and lymph node compartments represent average values from all metastatic tumors simulated and lymph node compartments, respectively. Solid curves show median values of virtual patients in each group. Shaded regions represent 95% confidence intervals. Colors represent response status. Patients with complete/partial response and stable disease were considered as responders.

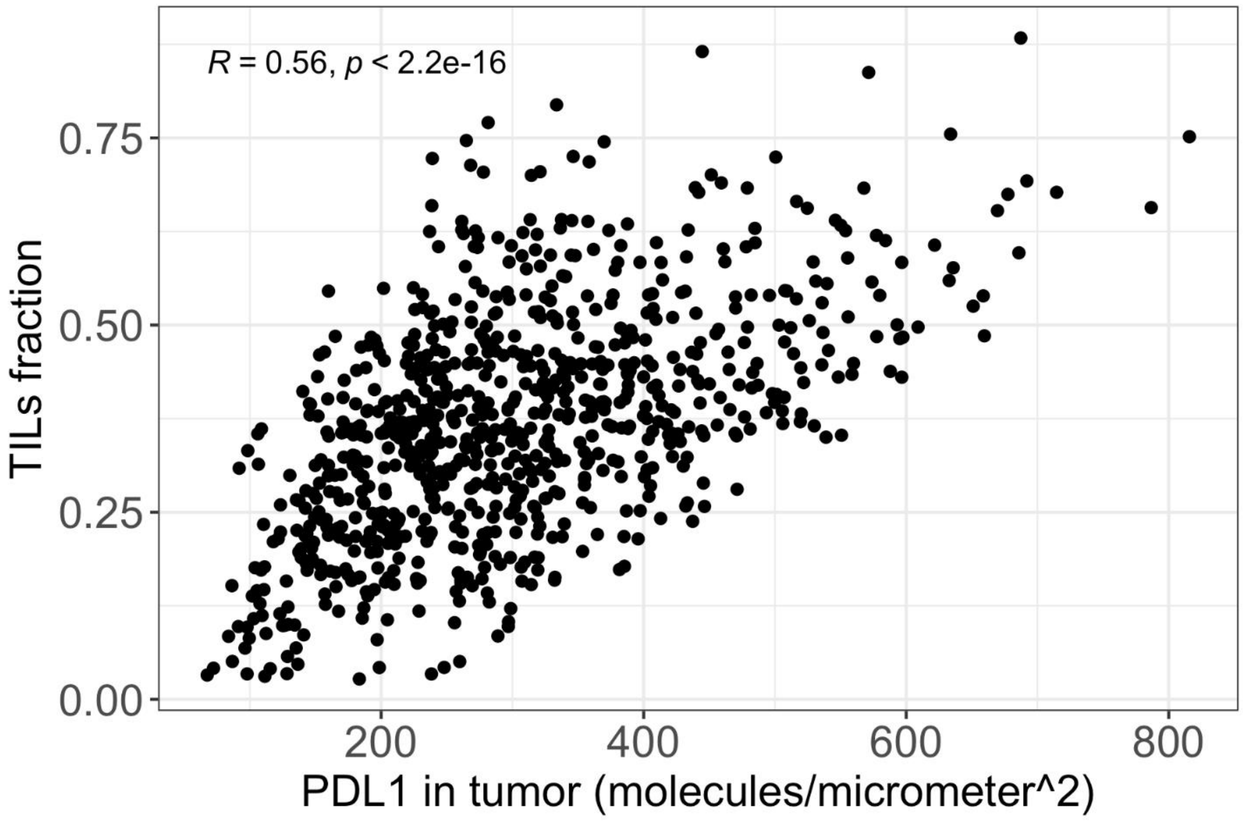

Figure S6: Correlation between PD-L1 in tumor and fraction of tumor infiltrating lymphocytes (TILs) in the virtual patients. Each dot indicates a virtual patient. R=correlation coefficient.

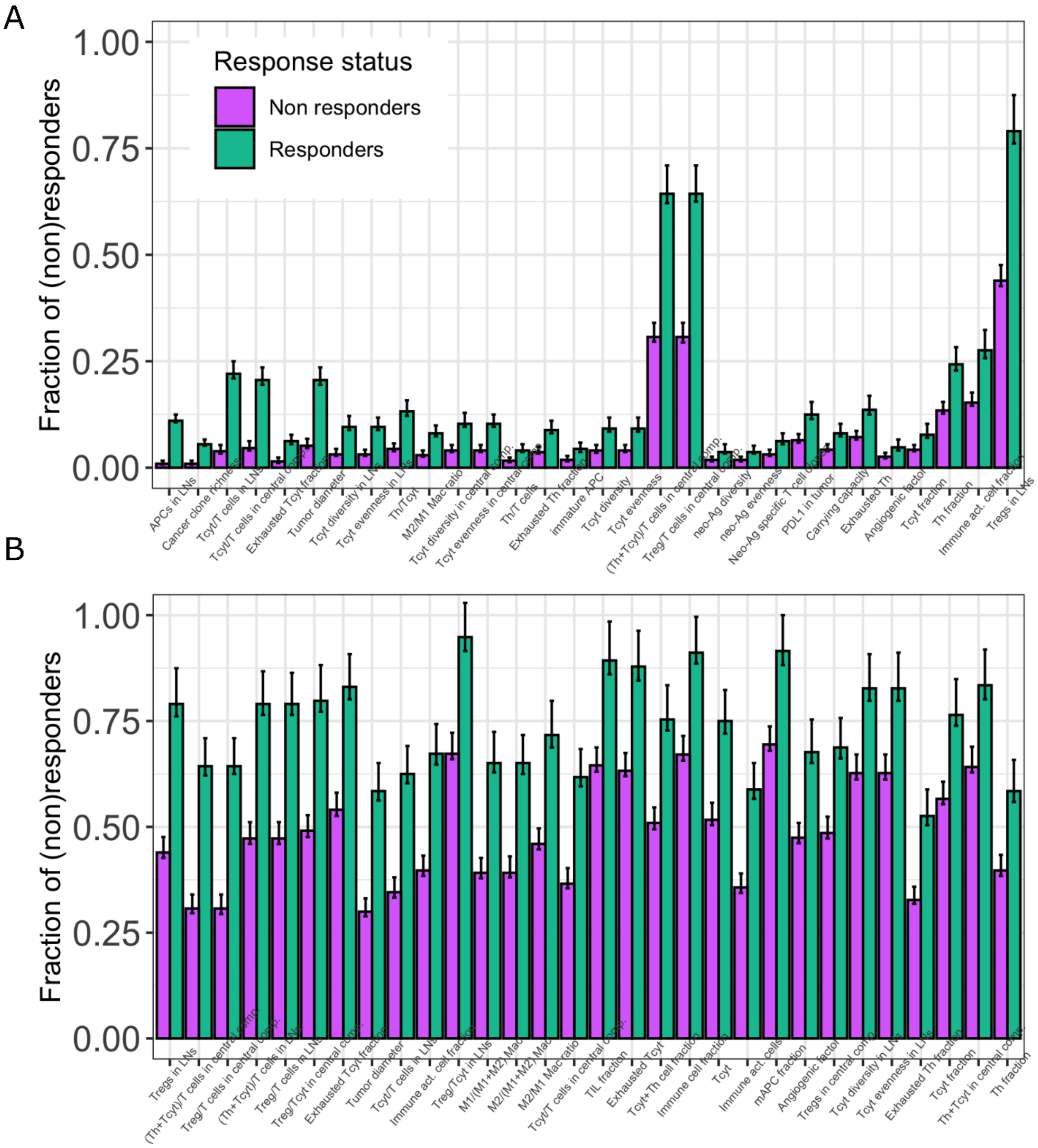

Figure S7: Fraction of responders and non-responders in virtual patient subgroups chosen based on best 30 individual biomarkers chosen based on response probability **(A)** or RIS **(B)**. Biomarkers are sorted based on decreasing response probability for panel A or RIS for panel B as shown in Figure 5. Error bars represent 95% confidence intervals calculated by bootstrapping. Ag: Antigen; TIL: Tumor infiltrating lymphocytes; APC: Antigen presenting cells (mature and immature), mAPC: Mature antigen presenting cells; LN: Lymph nodes; Tcyt: Cytotoxic T cells; Th: Helper T cells; Tregs: Regulatory T cells; Mac: Macrophages.

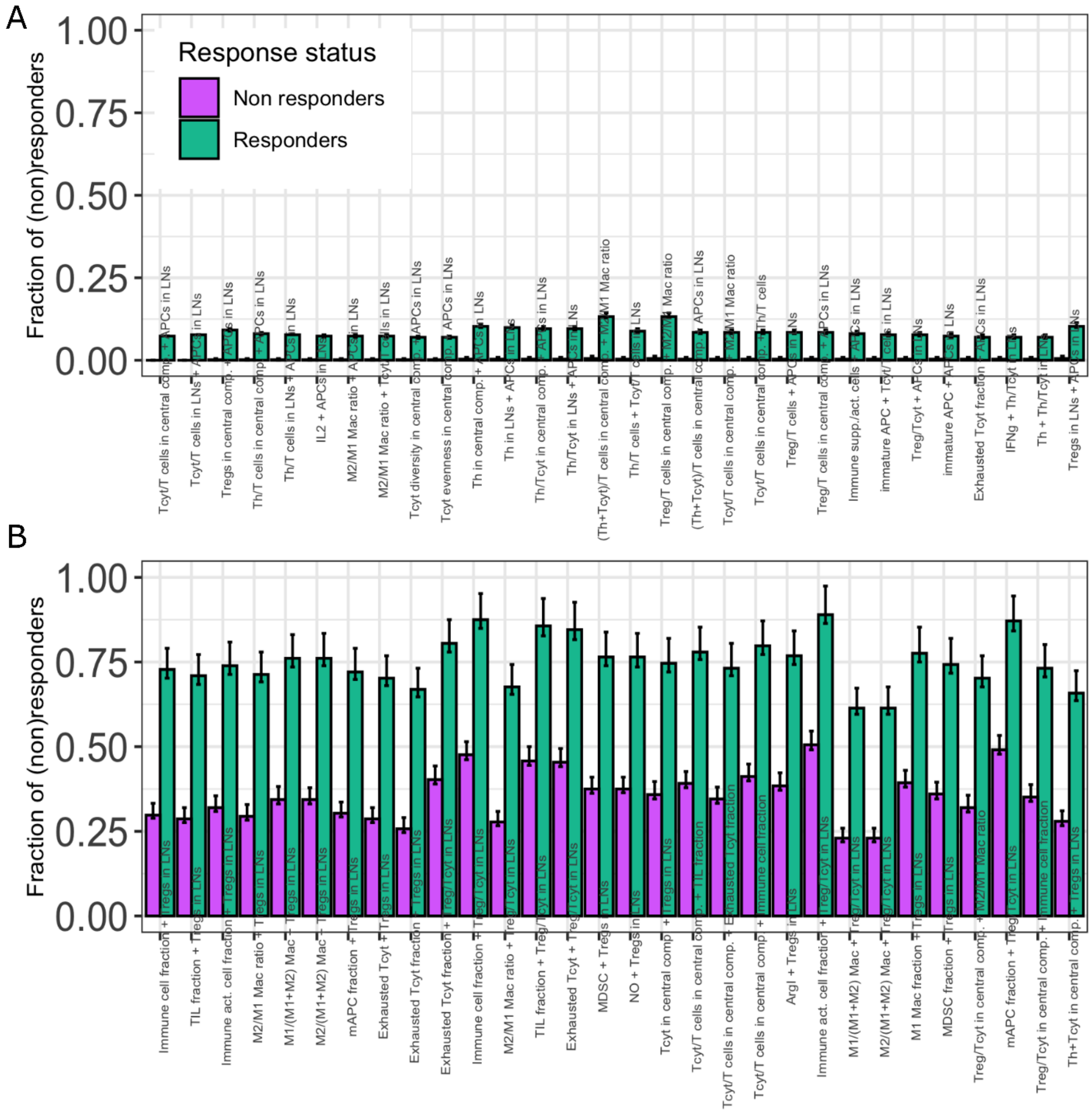

Figure S8: Fraction of responders and non-responders in virtual patient subgroups chosen based on best 30 biomarker combinations chosen based on response probability **(A)** or RIS **(B)**. Biomarker combinations are sorted in the sequence of decreasing response probability for panel A or RIS for panel B as shown in Figure 6. Error bars represent 95% confidence intervals calculated by bootstrapping. Ag: Antigen; TIL: Tumor infiltrating lymphocytes; APC: Antigen presenting cells (mature and immature); mAPC: Mature antigen presenting cells; LN: Lymph nodes; Tcyt: Cytotoxic T cells; Th: Helper T cells; Tregs: Regulatory T cells; Mac: Macrophages; IFNg: Interferon-γ; ArgI; Arginase-I; NO: Nitric oxide; MDSC: Myeloid-derived suppressor cells.

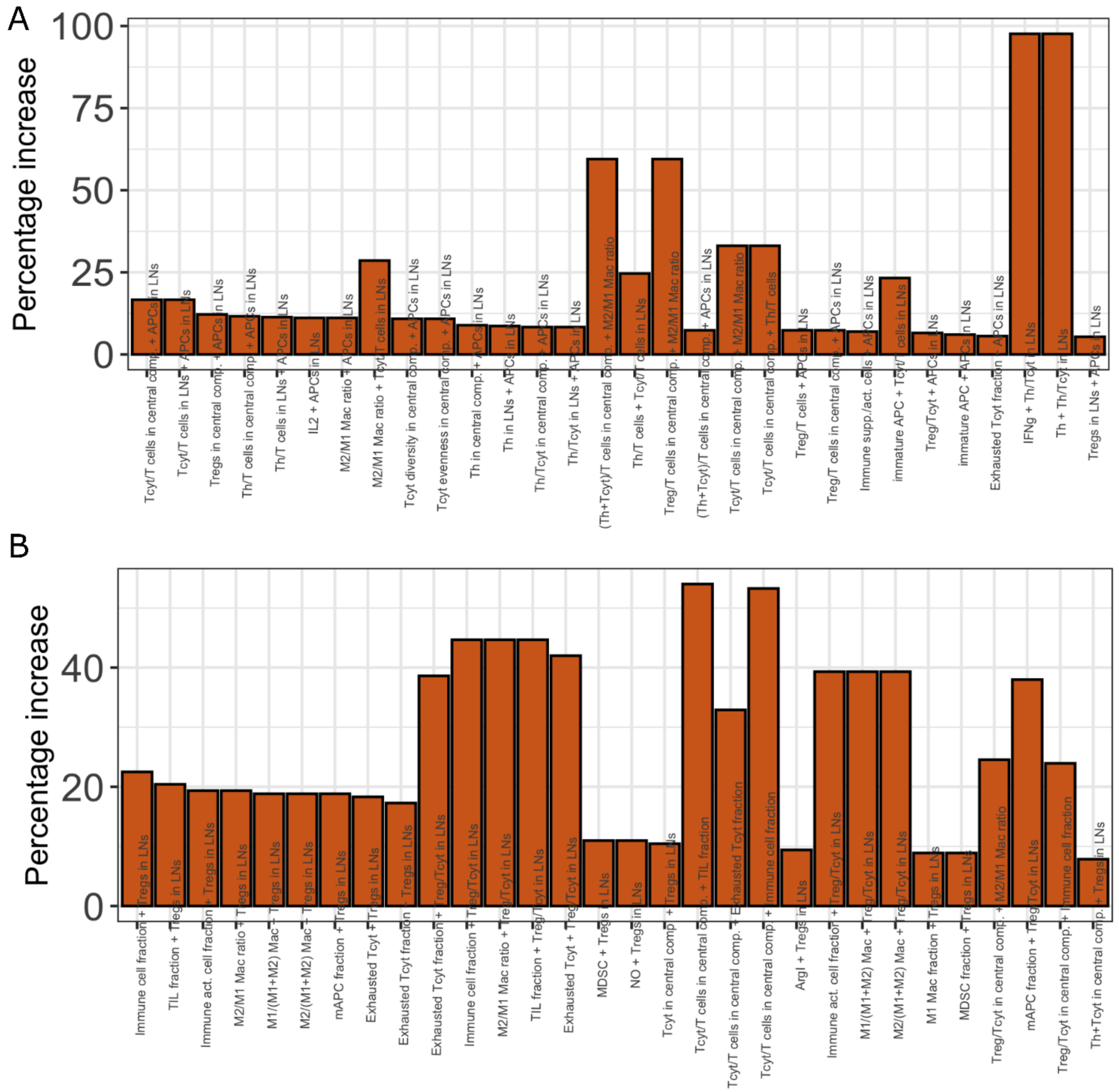

Figure S9: Percentage increase in response probability **(A)** or RIS **(B)** for biomarker combinations compared to the best individual biomarker in the combination. Biomarker combinations are sorted in the sequence of decreasing response probability for panel A or RIS for panel B as shown in Figure 6 and S8. Ag: Antigen; TIL: Tumor infiltrating lymphocytes; APC: Antigen presenting cells (mature and immature); mAPC: Mature antigen presenting cells; LN: Lymph nodes; Tcyt: Cytotoxic T cells; Th: Helper T cells; Tregs: Regulatory T cells; Mac: Macrophages; IFNg: Interferon-γ; ArgI; Arginase-I; NO: Nitric oxide; MDSC: Myeloid-derived suppressor cells.

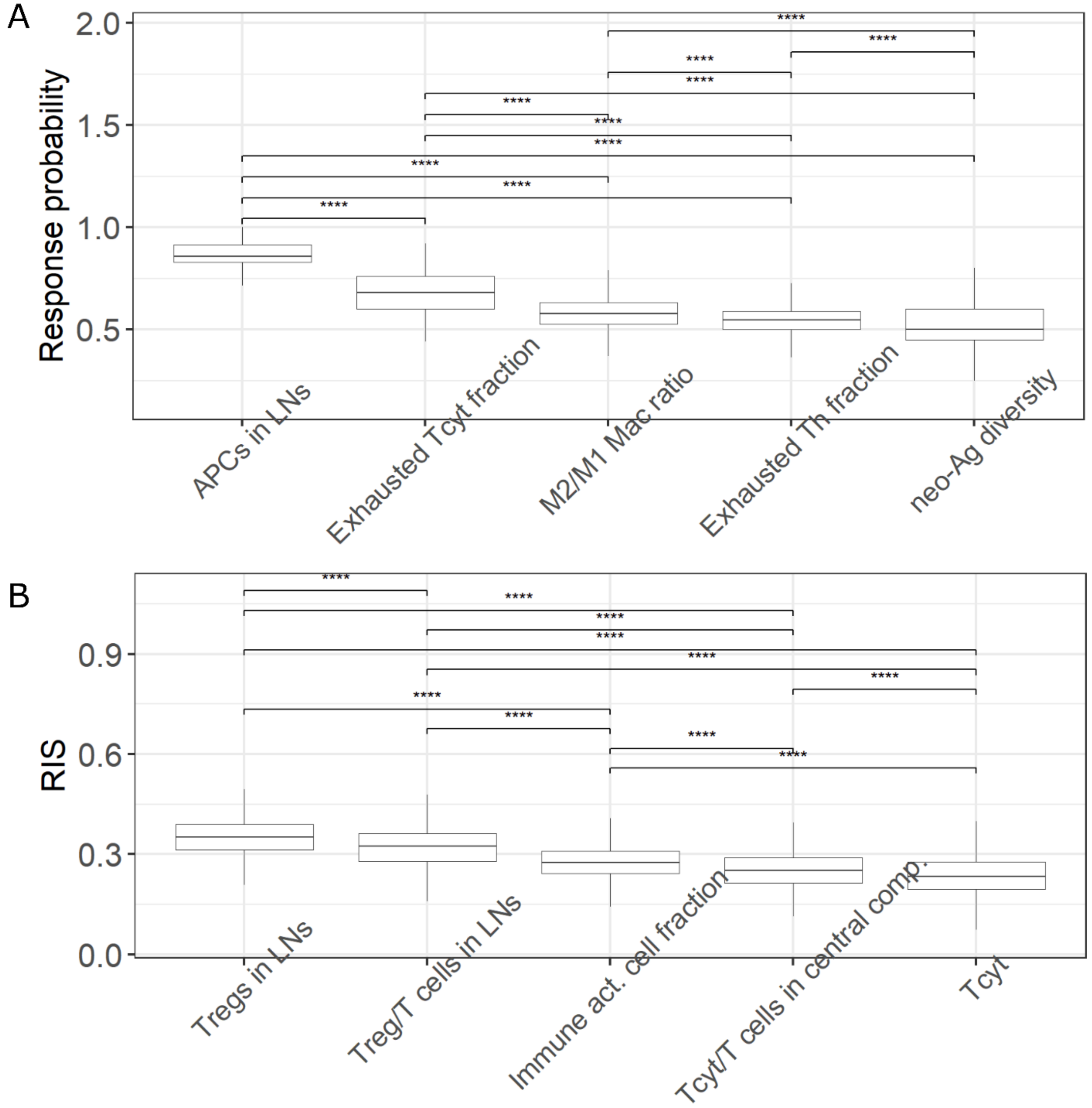

Figure S10: Statistical significance between selected biomarkers among top 20 individual biomarkers identified based on response probability **(A)** or RIS **(B)**. Ag: Antigen; APC: Antigen presenting cells (mature and immature); LN: Lymph nodes; Tcyt: Cytotoxic T cells; Th: Helper T cells; Tregs: Regulatory T cells; Mac: Macrophages. Statistical significance was computed by Wilcoxon test (**** = $p < 0.0001$).

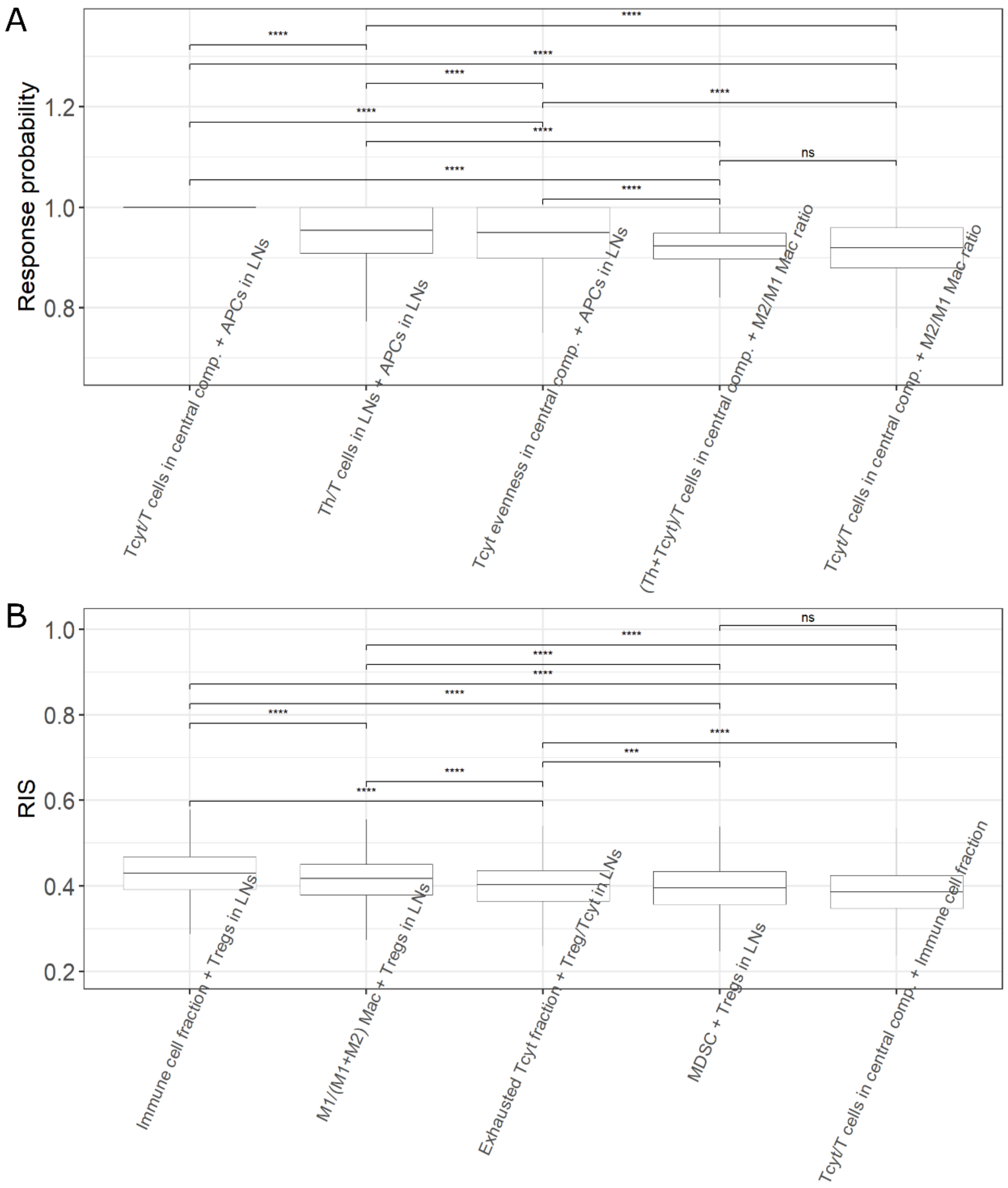

Figure S11: Statistical significance between selected biomarkers among top 20 biomarker combinations identified based on response probability **(A)** or RIS **(B)**. APC: Antigen presenting cells (mature and immature); LN: Lymph nodes; Tcyt: Cytotoxic T cells; Th: Helper T cells; Tregs: Regulatory T cells; Mac: Macrophages; MDSC: Myeloid-derived suppressor cells. Statistical significance was computed by Wilcoxon test (**** = p < 0.0001; *** = p < 0.001; ns=not significant).

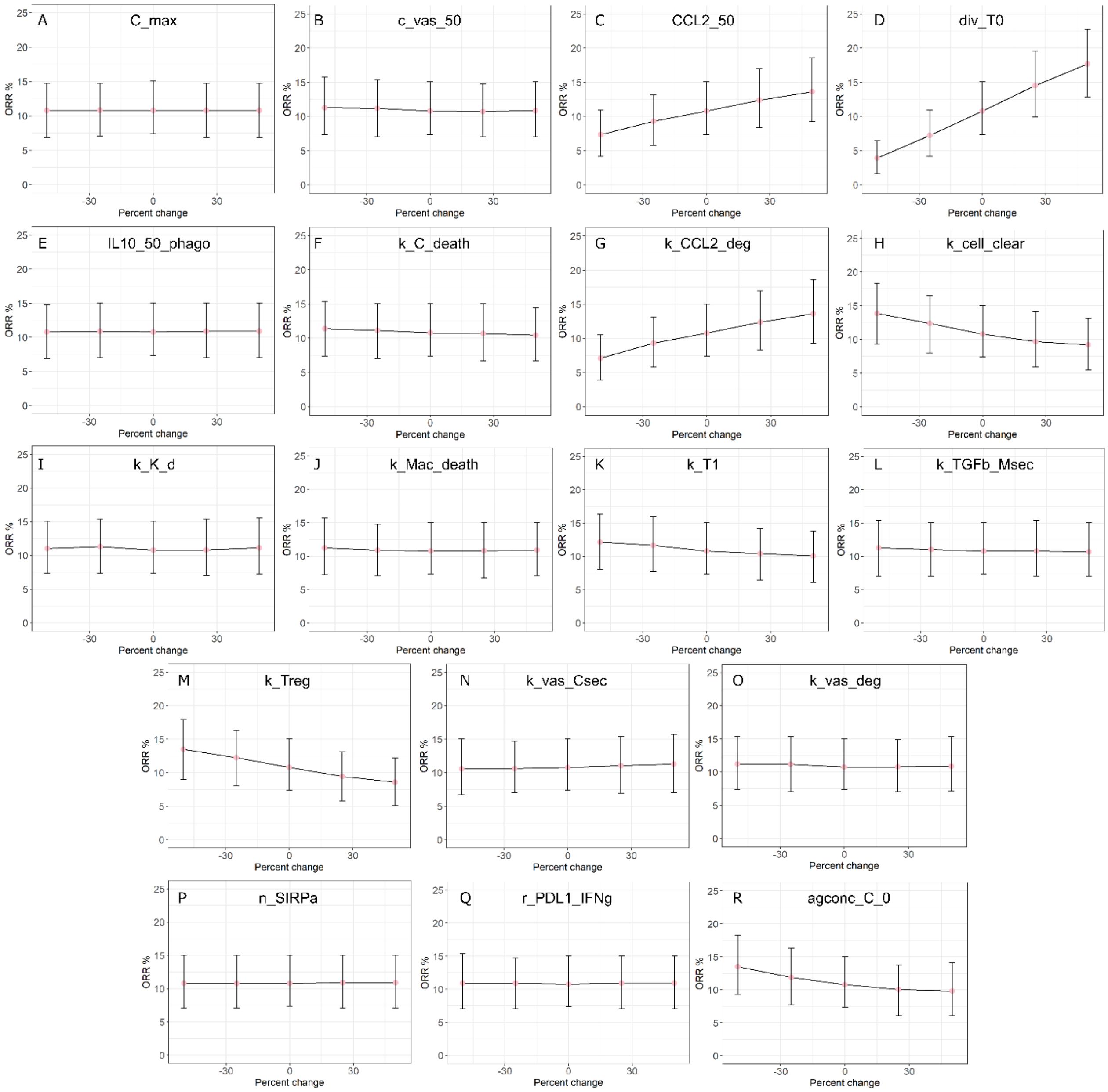

Figure S12: Sensitivity of ORR (objective response rates) to perturbations in model parameter values. (A) Carrying capacity (C_max), **(B)** Half-maximal concentration of angiogenic factor for the growth of carrying capacity (c_vas_50), **(C)** Half-maximal CCL2 level for MDSC recruitment (CCL2_50), **(D)** T cell diversity (div_T0), **(E)** Half-maximal IL10 level for phagocytosis by macrophages (IL10_50_phago), **(F)** Death rate constant of cancer cells by innate immune cells (k_C_death), **(G)** Rate constant for the degradation of CCL2 (k_CCL2_deg), **(H)** Rate constant of dead cell clearance from the tumor compartments (k_cell_clear), **(I)** Inhibition rate constant of tumor vasculature (k_K_d), **(J)** Death rate constant of macrophages (k_Mac_death), **(K)** Rate constant of T cell exhaustion by cancer cells (k_T1), **(L)** Secretion rate constant of TGF-β by macrophages (k_TGFb_Msec), **(M)** Rate constant of T cell death by Tregs

(k_Treg), **(N)** Secretion rate constant of angiogenic factor by cancer cells (k_vas_Csec), **(O)** Degradation rate constant of angiogenic factors (k_vas_deg), **(P)** Hill coefficient for CD47/SIRPα mediated inhibition of phagocytosis by macrophages (n_SIRPa), **(Q)** Fold increase in PDL1 expression by IFN-γ (r_PDL1_IFNg) and **(R)** Concentration of self-antigen in cancer cells (agconc_C_0).

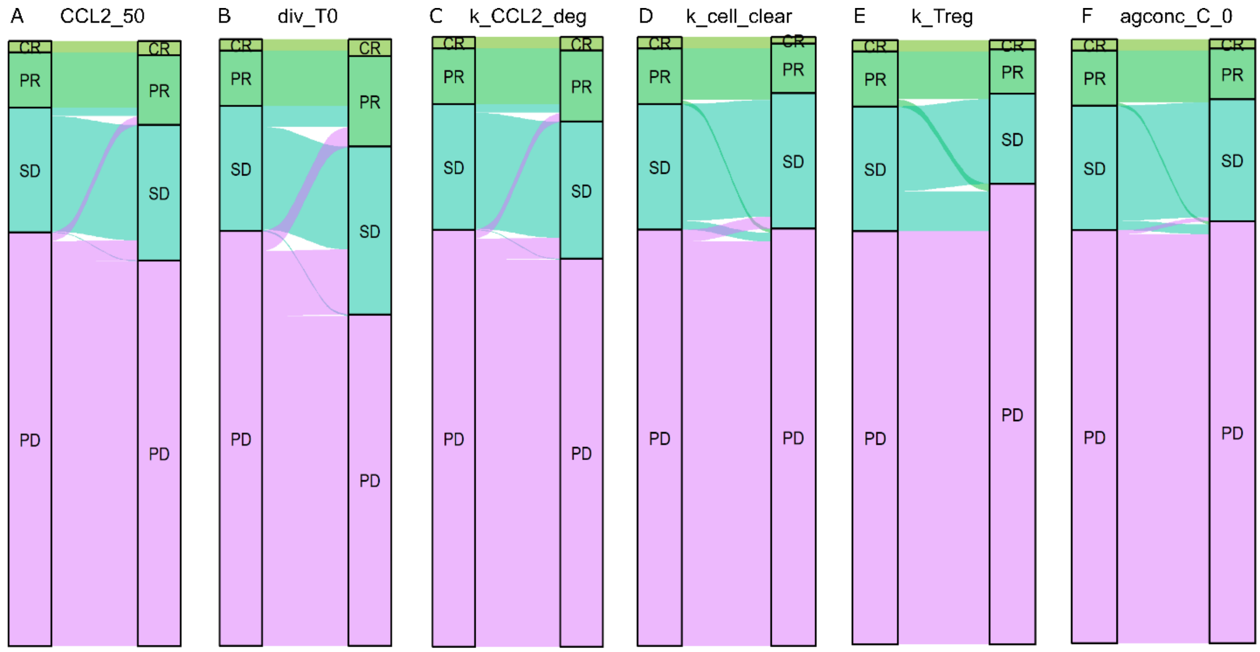

Figure S13: Sankey diagram showing the alterations in the response status of virtual patients upon parameter perturbations. (A) Half-maximal CCL2 level for MDSC recruitment (CCL2_50), **(B)** T cell diversity (div_T0), **(C)** Rate constant for the degradation of CCL2 (k_CCL2_deg), **(D)** Rate constant of dead cell clearance from the tumor compartments (k_cell_clear), **(E)** Rate constant of T cell death by Tregs (k_Treg), and **(F)** Concentration of self-antigen in cancer cells (agconc_C_0). In each panel, bar on the left shows the response status of virtual patients with reference parameter values (no perturbations) and the bar on the right shows the response status after 50% increase in the parameter values with respect to the reference parameter value. Reference parameter values are provided in the supplemental file Data S1.

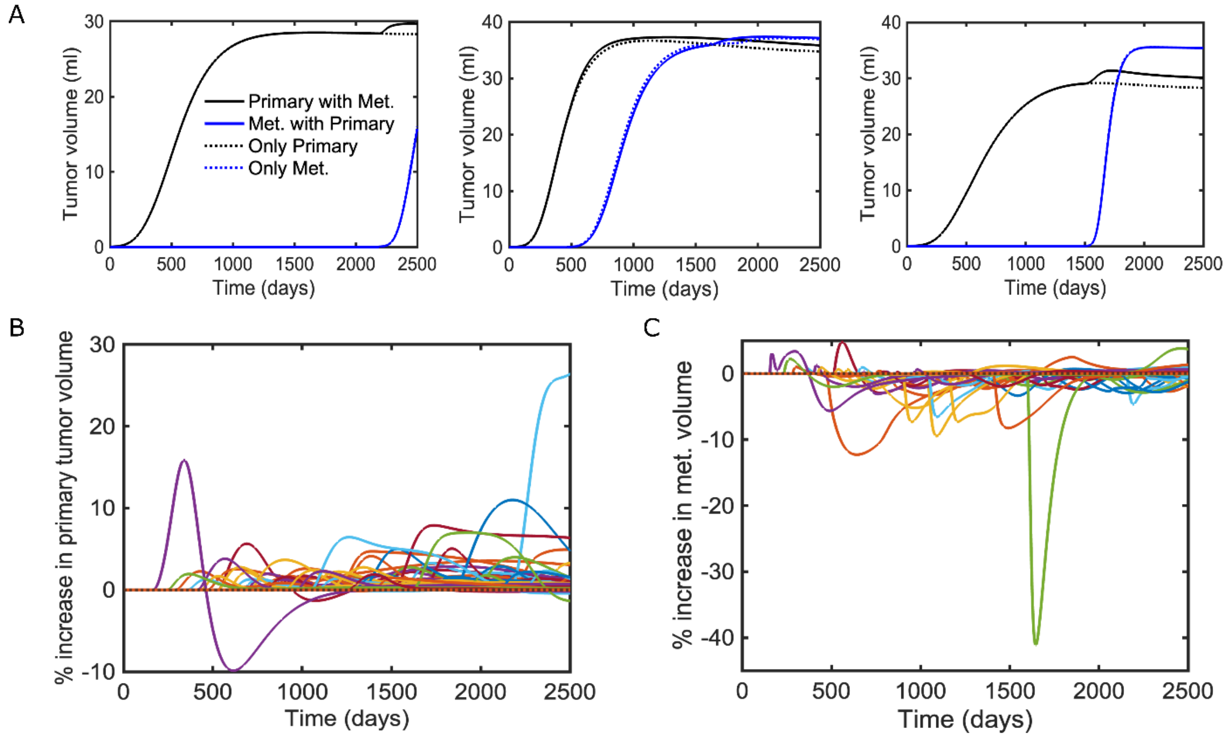

Figure S14: Interaction between primary tumor and a metastatic tumor in the QSP model. **(A)** Primary and metastatic tumor volumes in three representative virtual patients; Solid lines show the volume of primary tumor (black) or metastatic tumor (blue) in the presence of both tumors within a virtual patient. Dotted lines show the volume of primary tumor in the absence of metastatic tumor and vice versa, **(B)** Percent change in the volume of primary tumor in the presence of metastatic tumor, **(C)** Percent change in the volume of metastatic tumor in the presence of primary tumor. Each curve represents a virtual patient among 100 virtual patients simulated.

Data S1. (separate file)
Model reactions and parameters

Data S2. (separate file)
Results of *in-silico* biomarker analysis
